# Biopterin metabolism and nitric oxide recoupling in cancer

**DOI:** 10.3389/fonc.2023.1321326

**Published:** 2024-02-26

**Authors:** Gene Chatman Clark, Alan Lai, Aashri Agarwal, Zheng Liu, Xiang-Yang Wang

**Affiliations:** ^1^Department of Biochemistry, Virginia Commonwealth University, Richmond, VA, United States; ^2^School of Medicine, Virginia Commonwealth University, Richmond, VA, United States; ^3^Cornell University, Ithaca, NY, United States; ^4^Department of Human Molecular Genetics, Virginia Commonwealth University, Richmond, VA, United States; ^5^Massey Cancer Center, Virginia Commonwealth University, Richmond, VA, United States; ^6^Institute of Molecular Medicine, Virginia Commonwealth University, Richmond, VA, United States

**Keywords:** tetrahydrobiopterin, radiotherapy, nitric oxide synthase, immunotherapy, immunometabolism, vascular normalization, cancer, S-nitrosylation

## Abstract

Tetrahydrobiopterin is a cofactor necessary for the activity of several enzymes, the most studied of which is nitric oxide synthase. The role of this cofactor-enzyme relationship in vascular biology is well established. Recently, tetrahydrobiopterin metabolism has received increasing attention in the field of cancer immunology and immunotherapy due to its involvement in the cytotoxic T cell response. Past research has demonstrated that when the availability of BH4 is low, as it is in chronic inflammatory conditions and tumors, electron transfer in the active site of nitric oxide synthase becomes uncoupled from the oxidation of arginine. This results in the production of radical species that are capable of a direct attack on tetrahydrobiopterin, further depleting its local availability. This feedforward loop may act like a molecular switch, reinforcing low tetrahydrobiopterin levels leading to altered NO signaling, restrained immune effector activity, and perpetual vascular inflammation within the tumor microenvironment. In this review, we discuss the evidence for this underappreciated mechanism in different aspects of tumor progression and therapeutic responses. Furthermore, we discuss the preclinical evidence supporting a clinical role for tetrahydrobiopterin supplementation to enhance immunotherapy and radiotherapy for solid tumors and the potential safety concerns.

## Introduction

**Figure 1:**
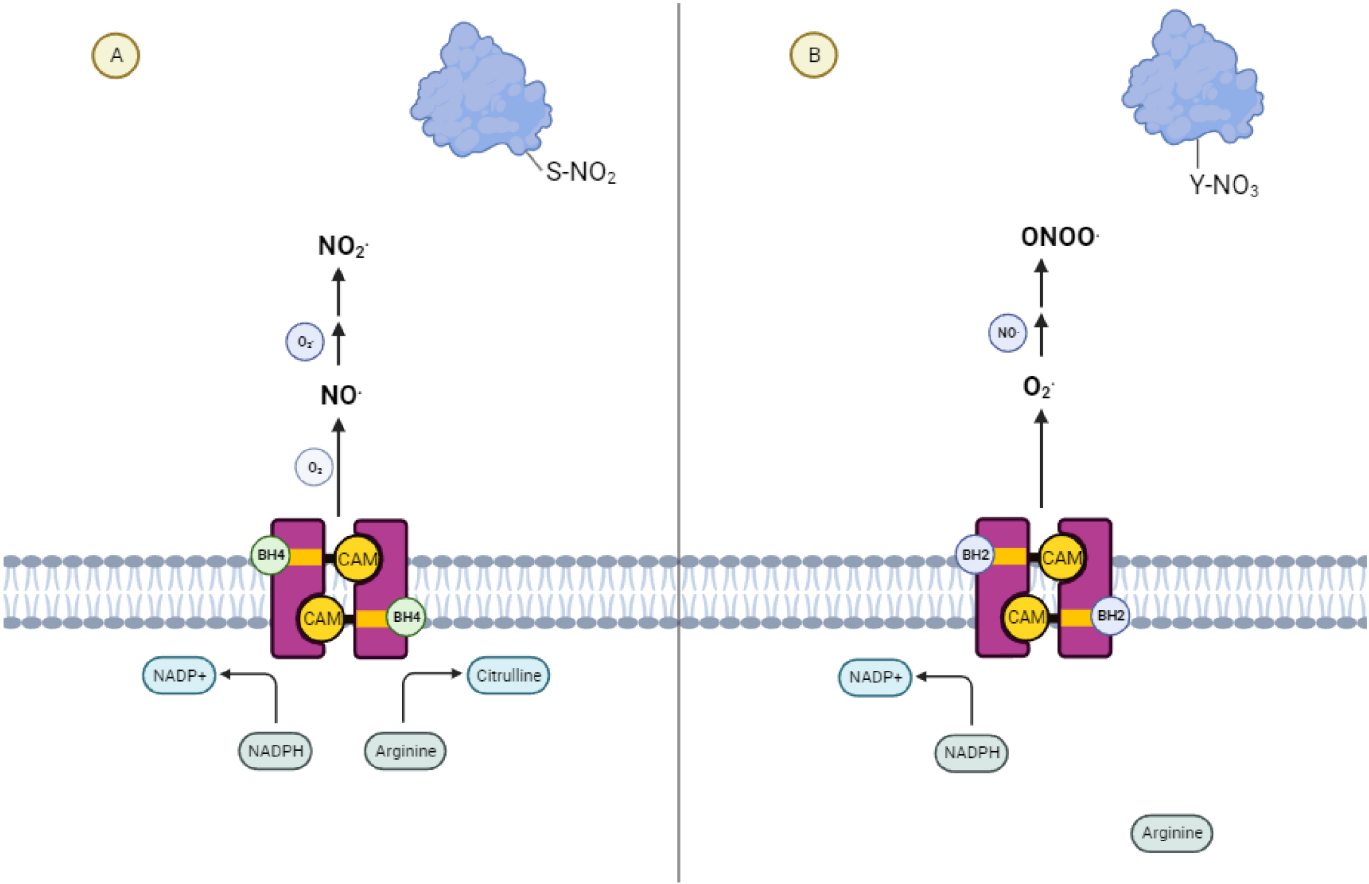
NOS Uncoupling and the Generation of Reactive Nitrogen Species **(A)** Coupled Nitric Oxide Synthase activity leads to signaling dominated by S-nitrosylative reactions mediated by nitric oxide. **(B)** Uncoupled Nitric Oxide Synthase activity leads to signaling dominated by nitrosative reactions mediated by peroxynitrite.

Nitric oxide synthase (NOS) exhibits two alternative activities depending on the relative availability of its cofactor tetrahydrobiopterin (BH4) (1). (**Figure 1**) In the presence of sufficient BH4, NOS catalyzes the production of NO and citrulline from the amino acid arginine. A necessary component of this process is the transfer of NADPH-derived electrons by BH4 to the ferrous dioxygen complex within the NOS active site (2, 3). When the availability of BH4 is low, as it is in chronic inflammatory conditions (4) and tumors (5), electron transfer in the active site becomes uncoupled from the oxidation of arginine, resulting in the production of a superoxide radical (6, 7). In the presence of trace amounts of NO, these two species react to form the short-lived, tyrosine-nitrating radical, peroxynitrite. This further reduces BH4 levels by direct oxidation (1, 8–10), producing a destructive feed-forward loop (11, 12). Due to this evolutionarily conserved (13) phenomenon of “NOS uncoupling,” the restriction of this cofactor is reinforced upon depletion of local BH4 until NOS activity ceases.

Due to its capacity for antigen-specific cell killing and immune memory, the T lymphocyte is a major focus in studies on therapeutic anti-tumor immunity. T cells recognize peptides derived from intracellularly degraded proteins and loaded onto Major Histocompatibility Complex (MHC) proteins on the cell surface in a process commonly referred to as antigen presentation (14). Two broad classes of T cells exist and are necessary for anti-tumor immunity (15, 16). T cells that express the co-receptor CD4 recognize antigens loaded onto MHC-II proteins on the surfaces of APCs. These “helper” T cells organize the adaptive immune response to tumors by secreting pro-inflammatory cytokines and chemokines (16). T cells that carry the co-receptor CD8 on the other hand recognize peptides loaded onto MHC-I molecules on the surfaces of cancer cells and carry out cell killing by secreting cell toxins or presenting cancer cells with FAS Ligand (15). CD8^+^ T cells can also be activated by MHC-I^+^ dendritic cells in a process called cross-presentation, which is crucial for generation of an effective anti-tumor immune response (17). CD4 and CD8 T cells are necessary for both the responses of tumors to immunotherapy and for the immunological memory required for durable responses to anti-cancer therapy (18, 19). Recently, it has been reported that ambient BH4 availability is a rate limiting factor required for the growth and proliferation of T cells after T cell receptor (TCR) engagement (20). Furthermore, pharmacological augmentation of BH4 levels has been demonstrated to enhance T cell function in multiple models including anti-tumor immunity (20). This suggests that mechanisms that alter BH4 metabolism within the tumor microenvironment (TME), such as NOS uncoupling, may play have a significant impact on immune evasion and the resistance of tumors to immunotherapy (14).

Considering the inducible nature of NOS expression within the tissues of the body (21), NOS-mediated BH4 consumption may also, ostensibly, limit the availability of this cofactor for other BH4-dependent enzymes such as alkylglycerol monooxygenase (AGMO) and the three aromatic amino acid hydroxylases: phenylalanine hydroxylase, tyrosine hydroxylase, and tryptophan hydroxylase. As reviewed elsewhere (22–24), these enzymes also make contributions to the immunosuppressive TME, making this an attractive area for further study (22–24). NOS uncoupling also has significant tissue level effects beyond the consumption of BH4. NO plays an essential role in the genesis and maturation of nascent vessels as well as the quiescence of mature vasculature (25, 26). As such, its absence in the TME contributes to the irregular structure and dysfunction that is common to tumor vasculature (27–30). This altered vasculature is both a hallmark of tumorigenesis in its own right and leads to therapeutic resistance through multiple mechanisms (31). Thus, exogenous BH4 supplementation may alter the characteristics of TME to improve traditional anti-cancer therapy (32), and also potentially make them more susceptible to immune attack (20, 33–35).

NOS can be effectively “recoupled” by supplementation with BH4 or its precursor sepiapterin. The therapeutic effect of NOS recoupling on endothelial dysfunction in hypertension, diabetes, and myocardial infarction is well recognized (36–40). However, the therapeutic potential of NOS recoupling in the context of cancer has only just begun to be explored. It has been demonstrated that the BH4/BH2 ratio is low in multiple *in vivo* tumor models and patient biopsies (5, 27, 32). Reconstitution of coupled NOS signaling with sepiapterin resulted in vasculature normalization, alleviating tumor hypoxia and enhancing the efficacy of chemotherapy (5, 27, 32). Lastly, sepiapterin supplementation has the added benefit of enhancing cytotoxic T cell proliferation and activity *in vivo* (20). Considering the potential of BH4 supplementation to synergize with existing cancer therapies as well as the excellent and well-established safety of both sepiapterin and BH4 in humans (41–45), BH4 supplementation may become an important tool in the anti-cancer arsenal of the future. In this review, we set out to describe the relevance of local BH4 depletion to existing anti-cancer therapies such as radiotherapy and immune checkpoint blockade with a focus on molecular mechanisms.

## NOS uncoupling and BH4 depletion

Biopterins are reductive, enzymatic cofactors required by organisms in all kingdoms of life (13). BH4 is the fully reduced form of biopterin in mammals and its availability is tightly regulated in normal tissues by elegant, cell-type specific control of its synthetic enzymes. Multiple mechanisms of NOS uncoupling and BH4 depletion within tumors have been reported. First, persistent generation of high levels of reactive oxygen and nitrogen species (ROS/RNS) is a hallmark of tumorigenesis (46). BH4 is highly reductive and therefore easily oxidized to its non-productive metabolite 7,8-dihydrobiopterin (BH2) by free radicals such as superoxide or peroxynitrite. Because these two biopterin species bind to the active site of NOS with equal affinities (47), the abundance of BH4 relative to BH2 is a crucial determinant of NOS activity. Additionally, exogenous producers of ROS/RNS, such as radiation, have been demonstrated to initiate NOS uncoupling, leading to reduced BH4 metabolism (48). Second, BH4 levels are regulated by transcriptional control of its synthetic enzymes. In chronic inflammation, BH4 production is limited due to the downregulation of GTP cyclohydrolase-I (49, 50) (GCH-I), the rate limiting enzyme in BH4 synthesis as well as by multiple signaling factors such as the immunosuppressive cytokines IL-10 and TGF-β (13). On the other hand, proinflammatory stimuli such as TNFα or TCR ligation stimulate the expression of GCH-I in their respective cell types (13). This dichotomous regulation of BH4 production by anti-inflammatory and proinflammatory signaling supports a central role of this cofactor in the immune response. Because once NOS uncoupling is initiated by low BH4 levels, its activity directly results in further ROS/RNS-mediated depletion of BH4, the effects of any one of these initial stimuli may be perpetuated until NOS activity ceases or is re-coupled to NO production. This feed-forward loop may represent a molecular switch, flipped off and on by anti-inflammatory and proinflammatory signals, that governs the local availability of BH4 and its impact on the TME and other pathological states (51).

NOS uncoupling is also accompanied by a consequential shift in NO-mediated signaling, which is relevant both to tumor progression as well as the anti-tumor immune response. The first reported physiological role of NO was discovered when it was found to act as a secondary messenger that binds and activates soluble guanylate cyclase (sGC) in endothelial cells. However, in addition to the activation of sGC and its downstream effectors such as protein kinase G, NO also mediates signaling by oxidative S-nitrosylation of cysteine residues to form S-nitrosothiol groups within target proteins (52). This bona fide post-translational modification, functioning analogously to phosphorylation to activate or inactivate proteins by various physical mechanisms (52). Comparably to phosphorylation, this mechanism modulates proteins of all functional classes and is governed by enzymatic machinery that includes S-nitrosylases and denitrosylases, reflecting its long presence over the course of evolution (53–56). More on the functional role of this post-translational modification on the activation and inactivation of proteins relevant to the immune cascade is provided below.

As NOS becomes uncoupled, an accompanying shift from S-nitrosylation to tyrosine nitration occurs and is the direct product of the changing ratio between ambient NO and superoxide produced by the enzyme. Despite its unpaired electron, NO itself is relatively non-reactive, reacting only with transition metals and heme iron. However, NO can also be “activated” via interactions with low levels of superoxide to form more reactive intermediates such as peroxynitrite (ONOO^-^), nitrogen dioxide (NO_2_), and dinitrogen trioxide (N_2_O_3_), all of which are capable of interacting with nucleophiles like cysteine (57). Similar to NO, NO_2_ is lipophilic, migrating across cell membranes and S-nitrosylating proteins at a relative distance. ONOO^-^, on the other hand, is not lipophilic and reacts rapidly with CO_2_ to form a CO_2_-ONOO^-^ intermediate that quickly degrades into NO_2_ and N_2_O_3_ in the presence of high levels of NO. For this reason, when NO levels are higher than superoxide levels, as they are when NOS is coupled, cyclic-GMP cyclase, protein kinase G signaling, and nitrosative reactions predominate over the oxidative reactions caused by peroxynitrite (58). In contrast, when superoxide levels are higher than NO levels, as they are when NOS is uncoupled, oxidative reactions mediated by peroxynitrite predominate while NO signaling is inhibited due to its impaired production and rapid local consumption. Due to their charge and short half-life, the oxidative reactions mediated by peroxynitrite, such as tyrosine nitration, are relegated to the immediate area of superoxide production.

This dichotomy of RNS directed signaling has a profound impact on the character and biology of the TME. The baseline NO produced by coupled endothelial NOS plays an important role in maintaining vascular integrity via activation of endothelial sGC as well as the S-nitrosylation and silencing of inflammatory mediators such an NF-*κ* (59–61). The consequence of these effects is a quiescent and functional vasculature. When NOS is uncoupled, as it often is in the TME, peroxynitrite becomes the predominant RNS species produced. This metabolite exhibits a much different chemistry, preferring to react with immediately available tyrosine groups on nearby proteins, resulting in a relatively long-lived Tyr-nitration. The effect of Tyr-nitration is analogous to Tyr-phosphorylation and it’s effects are different for different residues and proteins. Although this mechanism is less well studied, peroxynitrite mediated Tyr-nitration and degradation of I*κ*α is an example, and coupled with the loss of NF-*κ* silencing S-nitrosylation, has been suggested to lead to self-perpetuating and potentially unresolved vascular inflammation characteristic of tumors tissues (62, 63). In the setting of the aforementioned T cell dysfunction that comes with NOS mediated BH4 depletion, these effects of NOS uncoupling may greatly contribute to the pro-tumorigenic character of the TME by facilitating immune escape, the development of dysfunctional tumor vasculature, and the propagation of tumor-promoting inflammation.

## BH4 metabolism in tumorigenesis

### BH4 and immune escape

Although BH4 is necessary for multiple cellular processes, the availability of this essential cofactor is a key determinant of immune cell activity (20, 34, 35). Of the cells of the immune system, the activity of the T lymphocyte is especially susceptible to restriction of BH4, making its control a crucial factor in the adaptive immune response. Since NOS activity is often associated with local BH4 availability, NOS uncoupling within tumor vasculature may represent a novel pathway involved in tumor immune escape.

BH4 availability has been shown to act as a rate-limiting factor for T cell proliferation and activity in diverse models of immunity, including within solid tumors (20, 64). This acceleration in T cell proliferation may occur due to increased iron-redox recycling of T cell mitochondrial cytochrome c which otherwise results in defective oxidative phosphorylation and superoxide production upon TCR stimulation (20, 35). It has been reported that expression of GCH1, the rate-limiting enzyme in BH4 production, is enhanced upon TCR stimulation and its inhibition antagonizes T cell proliferation (13). However, exogenous supplementation of BH4 has been demonstrated to enhance T cell activity in models of autoimmunity, suggesting that endogenous BH4 production by T cells does not support maximum activity and environmental BH4 is rate limiting (20). This supports the idea that the control of BH4 availability by NOS-expressing cells such as endothelial and myeloid cells may be a direct mechanism of impaired T cell activity both within and outside of the TME.

In this way, NOS uncoupling may have an indirect, albeit potent, effect on anti-tumor T cell activity by metabolizing BH4 to non-reducing metabolites. Therefore, supplementation of BH4 may counteract this effect both by supplying BH4 directly to proliferating T cells as well as recoupling NOS, alleviating further NOS-mediated BH4 consumption. Whether BH4 available for use by T cells is represented by free-floating, ambient BH4 or BH4 that is stored in the surrounding stroma has yet to be determined. However, it has been demonstrated that both BH4 production and sequestration occur in various cell types in response to pro-inflammatory cytokine stimulation (13).

In addition, much evidence exists that the restoration of NO signaling via recoupled NOS may support anti-tumor immunity as well. For instance, human T cells express NOS (65–69) and produce NO upon TCR activation, leading to enhanced activity of the TCR CD3-zeta chain, the adaptor kinase ZAP-70, as well as N-Ras/ERK pathways mediated by N-nitrolsylation (70–72). Furthermore, endogenous levels of NO, such as those produced by eNOS, promote the differentiation of Th1 T cells via a cGMP dependent pathway (73), while NOS uncoupling and oxidative stress support Th2 differentiation (74, 75). Additionally, FOXP3^+^ regulatory T cells have also been reported to be negatively regulated in a cGMP dependent manner although it is unclear if this is a direct result of NOS activity (76, 77).

**Figure 2:**
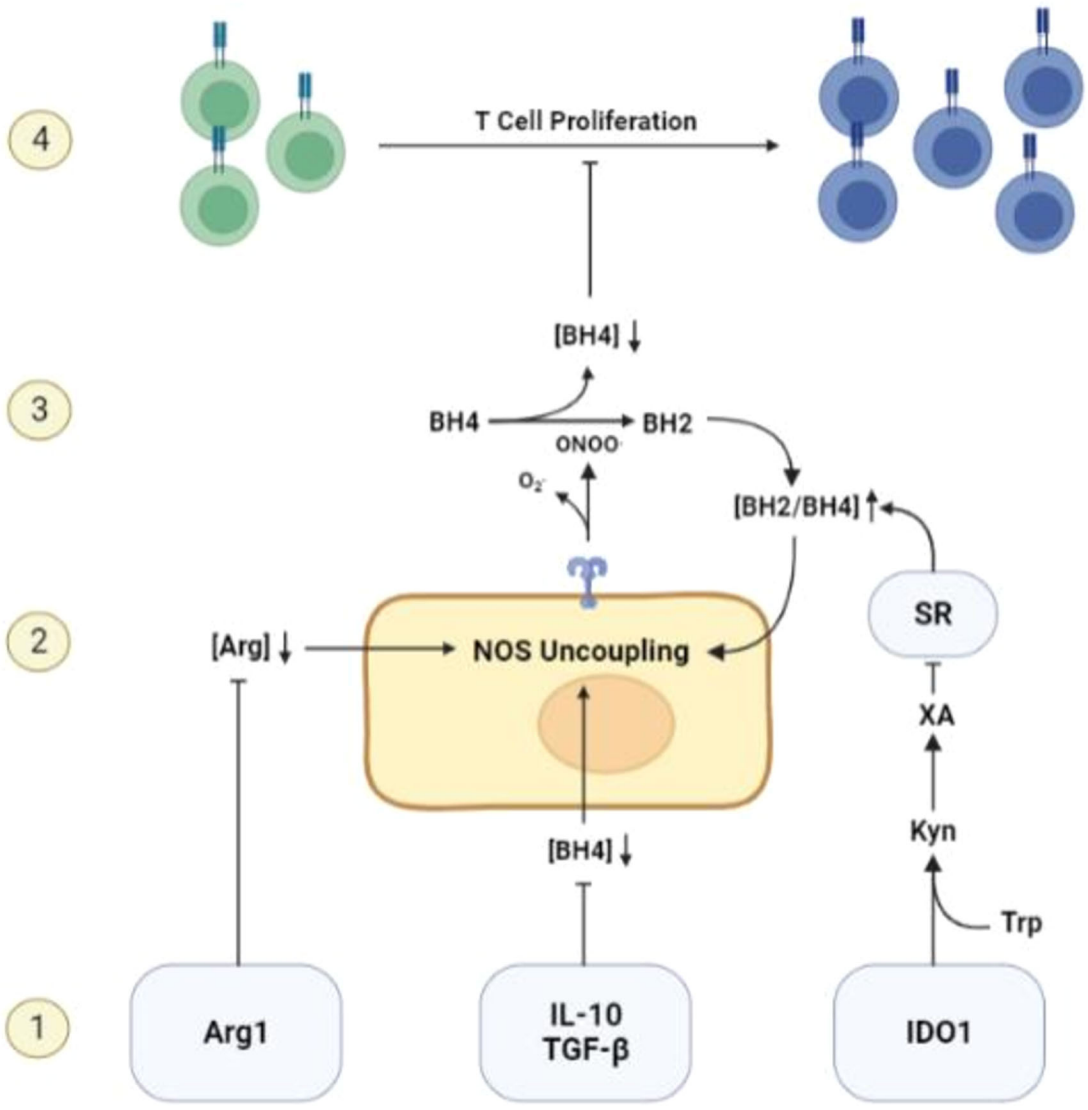
Mechanisms of BH4 depletion in the tumor microenvironment. 1) BH4 levels are suppressed by multiple common features of the TME including, but not limited to, the activity of Arg1 and IDO1, the expression of immunosuppressive cytokines such as IL-10 and TGF-β, and high levels of reactive oxygen species. 2) Low levels of ambient BH4 leads to uncoupling of NOS activity, leading to the production of ROS/RNS such as peroxynitrite, a potent oxidizer of BH4. 3) NOS-mediated BH4 depletion initiates a destructive feedforward loop, leading to local BH4 depletion and reinforcing uncoupled NOS activity. 4) local BH4 availability its limited and T cell proliferation is subsequently restricted.

Interestingly, two other classic immunosuppressive mechanisms in the TME, Arginase 1 (Arg1) and Indoleamine 2’3-Dioxygenase 1 (IDO1), can also cause BH4 restriction indirectly via NOS uncoupling (11, 78). Arg1 produces arginase, which breaks down ambient arginine that is required for T cell activity and proliferation. The metabolism of arginine by arginase expressing cells such as M2 macrophages and MDSCs, is a well-recognized mechanism causing T functional impairment within the TME (79). Arginine is also the ultimate electron acceptor in the coupled NOS reaction and required for the production of NO. In the absence of arginine, NOS activity will proceed by donating electrons directly to molecular oxygen, generating free radicals (13). This initiates the uncoupled NOS cascade, leading to ambient BH4 depletion and further restriction of T cell activity. IDO1, which causes T cell restraint by restricting ambient tryptophan (79), may also induce NOS-mediated BH4 depletion via a secondary metabolite of tryptophan metabolism, xanthuric acid. This metabolite has an inhibitory effect on sepiapterin reductase (SR), a necessary enzyme in the BH4 salvage pathway that catalyzes the production of BH4 from BH2 (80). The enhanced concentration of BH2 competes with BH4 for binding to NOS, leading to uncoupling of NOS activity and BH4 depletion (48). Considering the evolutionarily conserved phenomenon of NOS uncoupling as well as the sophisticated mechanisms governing its activation, NOS uncoupling may be a deliberate and physiological mechanism of T cell restriction that is co-opted by solid tumors to achieve immune escape (**Figure 2**).

In addition to depleting available BH4, peroxynitrite produced by uncoupled NOS has been demonstrated to antagonize T cell activity via more direct effects (81–83). This leads to both the direct nitration and inactivation of the TCR as well as nitration and inhibition of the chemokines CCL2 and CCL5, leading to decreased T cell accumulation within tumors (84–86). Taken together, these reports suggest that endogenous NO expression may contribute to immune surveillance in normal tissues and that NOS uncoupling is a necessary step in the development of immune escape. Highlighting the direct antagonistic effect of NOS uncoupling on T cell activity, de Sanctis et al. found that inhibiting ARG1 and NOS2 expression inhibited accumulation of nitro-Tyrosine moieties in T cells and sensitized pancreatic tumor models to attack by TERT specific CAR-T (33). In addition to modulating the negative effects of NOS uncoupling, restoring native NO production and downstream signaling by activated T cells has also been proposed as an attractive therapeutic strategy (87).

Conversely, it should be noted that some studies suggest that very high levels of NO, such as those produced by NO donors or potentially iNOS, may actually negatively regulate Th1 differentiation required for anti-tumor immunity by leading to the S-nitrosylation of key post-transcriptional regulators of Th1 cytokines and transcription factors (88–90). One report suggests that a FOXP3^-^ population of regulatory T cells may exist whose differentiation may be NO dependent (91). The discrepancy of these effects of NO on T cell phenotype may be explained by the time and dose dependence reported to characterize this pathway. Given the potential of BH4 supplementation to create high levels of NO via forced coupling of iNOS by immune cells within TME, their effects on immune functions need to further investigated.

### BH4 and tumor vasculature

One hallmark of tumorigenesis is the acquired ability of tumors to recruit and maintain their own blood supply. Many excellent reviews of our understanding of the mechanisms of tumor vascular recruitment exist (31, 92, 93) and these mechanisms will not be extensively reviewed here. Compared to vasculature elsewhere in the body, tumor vasculature is typically erratic and highly dysfunctional, characterized by blunt ends and inconsistent blood flow, resulting in insufficient tumor perfusion and high interstitial pressure (94–97). These characteristics lead to the poor penetration of chemotherapy (98–100) and can cause intra-tumoral hypoxia and an accumulation of acid (95, 101–104) which in turn antagonizes anti-tumor immunity by stimulating immune checkpoint expression (105, 106) and immunosuppressive myeloid cells. Activity of cytotoxic lymphocytes in the tumor may also be antagonized due to the abnormal nature of tumor vasculature (107–109). As a result, the potential of vasculature normalizing agents to alter the immunosuppressive TME is the subject of considerable ongoing research.

Coupled eNOS activity is a key mediator of vascular function throughout the body. eNOS mediated NO gradients are necessary for the recruitment of pericytes and smooth muscle cells required for vascular integrity (29). NO is also necessary for the migration of endothelial cells, the formation of new capillary structures (26, 110) such as tertiary lymphoid tissues, which are important for the proliferation of anti-tumor lymphocytes, and for the maturation of normal vasculature through the expression of Angiotensin 1 (25, 111–113). Finally, constitutive, physiologic levels of NO provided by eNOS is an important part of maintaining vascular quiescence. Underscoring the importance of NO signaling to vascular function, NOS recoupling with BH4 or sepiapterin has been demonstrated to stimulate neoangiogenesis and normalize the reactivity of dysfunctional endothelium in cardiovascular disease (38, 39, 114, 115).

The positive role of NO in regulating angiogenesis raises the question of whether recoupling NOS and restoring NO signaling has a beneficial effect on tumor biology. Within the TME, where NOS is uncoupled, NO signaling is absent and vascular inflammation is exacerbated by the effects of peroxynitrite. This leads to endothelial dysfunction, which is typical of tumor vasculature, and may partly explain the irregular structure and leakiness of the TME. Recoupling of endothelial NOS using oral sepiapterin leads to the normalization of tumor vasculature as demonstrated by the enhanced oxygenation of tumors, decreased hypoxia, and increase in pericyte coverage of tumor blood vessels (32). This is likely due to the inhibition of ROS/RNS (29) as well as the vasculature normalizing effect of physiological levels of NO (29, 116–118) enhancing tumor oxygenation and blood flow, thereby contributing to an enhanced efficacy of both radiotherapy and treatment with doxorubicin. Considering their potential synergy with immunotherapy, it is important to note that strategies that aim to restore physiological NO signaling by recoupling NOS appear to lead to vascular normalization as opposed to vascular disruption (27). This is contrary to the NOS inhibitor L-N^G^-nitroarginine which disrupts tumor vasculature causing hypoxia and tumor cell death (119–121). For interventions that rely on enhanced immunogenicity of the TME via vasculature modulation, normalization is more likely than vascular disruption to alleviate hypoxic immunosuppression. This is consistent with multiple studies demonstrating that the efficacy of immunotherapy could be enhanced by treatment with combination with anti-angiogenic agents at low doses, but that the use of high dose anti-angiogenic therapy antagonized immunotherapy due to the hypoxia induced by excessive vessel pruning (94, 96, 97).

### BH4 and tumor-promoting inflammation

Chronic inflammation is a hallmark of the TME, promoting tumor growth and invasion while paradoxically providing the means for escape from the adaptive immune response (122). While appropriate, physiological inflammation is an essential component of an anti-tumor immune response, the inappropriately prolonged, disordered inflammation associated with the TME actually serves as a barrier to T cell function and immune surveillance (123). It is partially for this reason that initiation of as many as a fifth of human cancers is linked to inflammation caused by chronic infection or exposure to irritants (124) as illustrated by the chemo-preventative effects of daily anti-inflammatory drug use on certain types of cancer.

**Figure 3:**
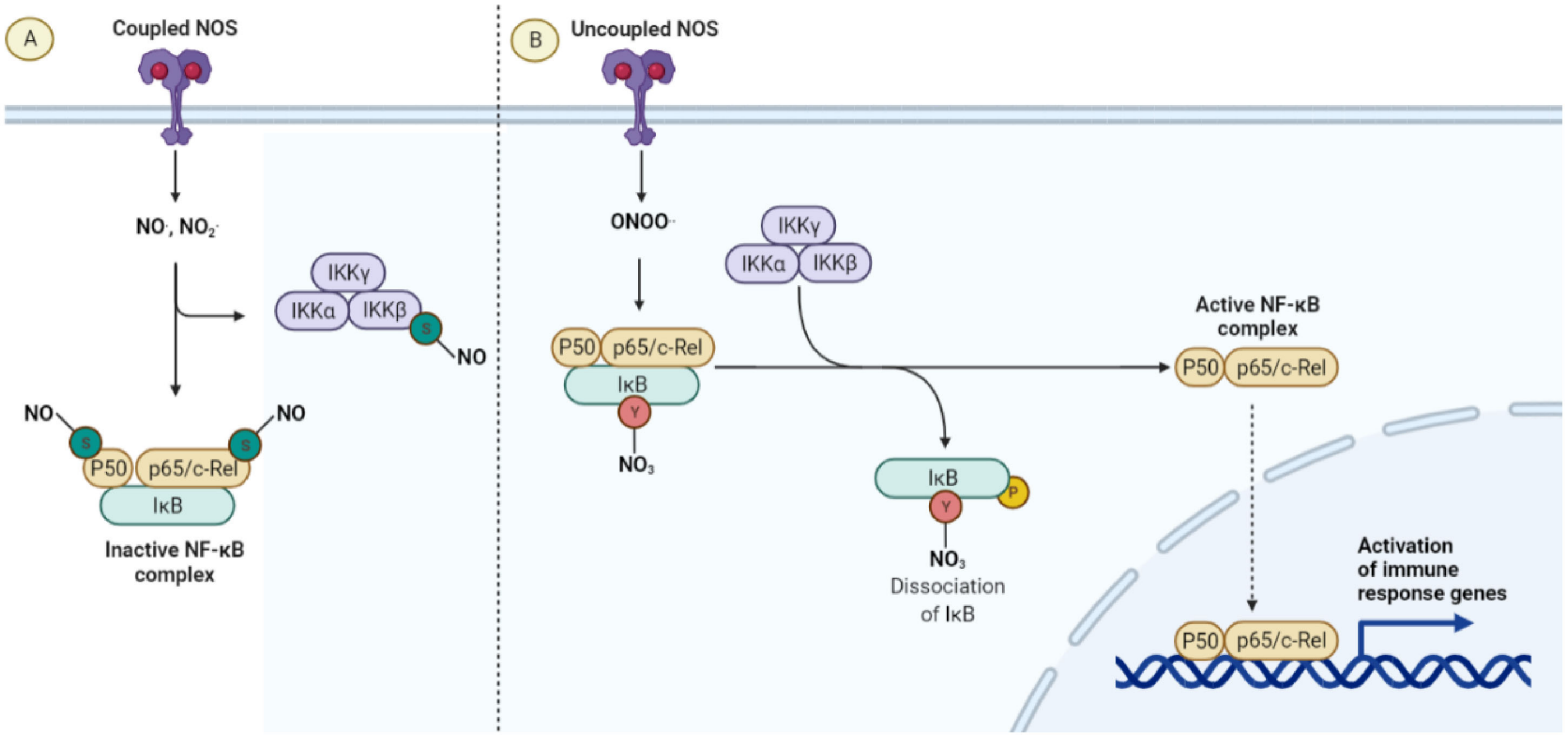
The anti-inflammatory effect of coupled NOS. **(A)** When NO is the predominant nitrogen species generated by NOS, anti-inflammatory S-nitrosylations of the NF-κB pathway lead to quiescence and normal endothelial function. **(B)** When NOS is uncoupled, peroxynitrite and ROS are the predominant species produced, leading to a loss of anti-inflammatory S-nitrosylation as well as the Y-nitration and inactivation of IκB.

As discussed above, coupled NOS activity plays a major role in maintaining vascular quiescence. This is due to the pleiotropic anti-inflammatory effects of NO-mediated signaling on the cells of the vasculature itself. Possibly the most important pathway regulated by NO is that of the master inflammatory transcription factor NF-*κ*B, (**Figure 3**) which is susceptible to silencing by S-nitrosylation. NO-dependent S-nitrosylation of the p65 subunit inhibits the transcriptional activity of NF-*κ*B in multiple cell types (59–61). The p50 subunit is also nitrosylated at a cysteine located in its DNA binding domain, resulting in an inhibition of inflammation (61). IKKβ has also been demonstrated to be negatively regulated by S-nitrosylation mediated by NO, resulting in the inhibition of NF-*κ*B activity (125). In addition to the loss of these silencing mechanisms, uncoupled NOS may directly activate this pathway via the Tyr-nitration and proteosome independent inhibition of I*k*Ba (126). Whether this additional positive regulation of the NF-*κ*B pathway occurs with NOS uncoupling *in vivo* remains to be seen.

Other mediators of inflammation are directly inhibited by NO mediated S-nitrosylation as well. CD40 is a member of the TNF receptor family, responsible for activating NF-*κ*B in response to stimulation with CD40L in what has been called the non-canonical NF-*κ*B pathway. S-nitrosylation of CD40 on the surface of resting monocytes and macrophages has been observed to inhibit signal transduction in response to CD40 activation, suggesting that denitrosylation may be a prerequisite for activation of this pathway (127). S-nitrosylation of S100A8 has also been suggested to be an important step in the resolution of inflammation by limiting the endothelial cell-lymphocyte interactions in circulation (128). AP-1 is a transcription factor responsible for cytokine production and proliferation in response to multiple stimuli. It is activated by a MAPK cascade resulting in the activation of the kinase c-Jun N-terminal Kinase (JNK) which phosphorylates and activates c-Jun, one of its components. JNK1 is susceptible to NO mediated S-nitrosylation at Cys-119, further contributing to the anti-inflammatory effect of NO (129, 130). Finally, iNOS itself is inhibited by S-nitrosylation (131, 132), suggesting that NOS uncoupling may be further perpetuated by an increase in iNOS expression under conditions that support superoxide production and BH4 depletion supporting the idea that NOS uncoupling may function as a molecular switch.

NO has also been recognized for its inflammation suppressing effects in quiescent tissues by inhibiting leukocyte adhesion to the vascular wall, preventing the extravasation of immune cells into the underlying tissue (133–135). More recently, it has been suggested that this may be due in part to the suppression of P-selectin expression in the vascular endothelium mediated by constitutive, eNOS dependent sGC activation (136). Previous reports demonstrated that inactivation of sGC is a critical step in the recruitment of myeloid cells to the colon after DSS treatment. It was found by our group that the recruitment of inflammatory macrophages and neutrophils to the colon could be inhibited by treatment with sepiapterin, an effect that was partially inhibited by a cGC inhibitor (4). The consequence of therapeutically augmenting BH4 levels within tumors was the resolution of NF-*κ*B driven inflammation. This resulted in the inhibition of tumor formation in a spontaneous colon cancer model (4, 5). This finding is relevant to this tumor type for which inflammation plays such a well-recognized role in progression as well as due to previous findings that suggest BH4 levels are low in human colorectal tumor biopsies when compared to normal tissue (32). All of these are important co-stimulators of T-cell mediated anti-tumor immunity and extravasation. However, the inappropriate, persistent activity of these inflammatory mediators leads to T cell dysfunction due to the effects of long-term inflammation characteristic of the TME. The various mechanisms of this effects are expertly reviewed elsewhere (123, 124, 137, 138).

## The Effects of BH4 supplementation on Cancer Therapies

### Immunotherapy

**Figure 4:**
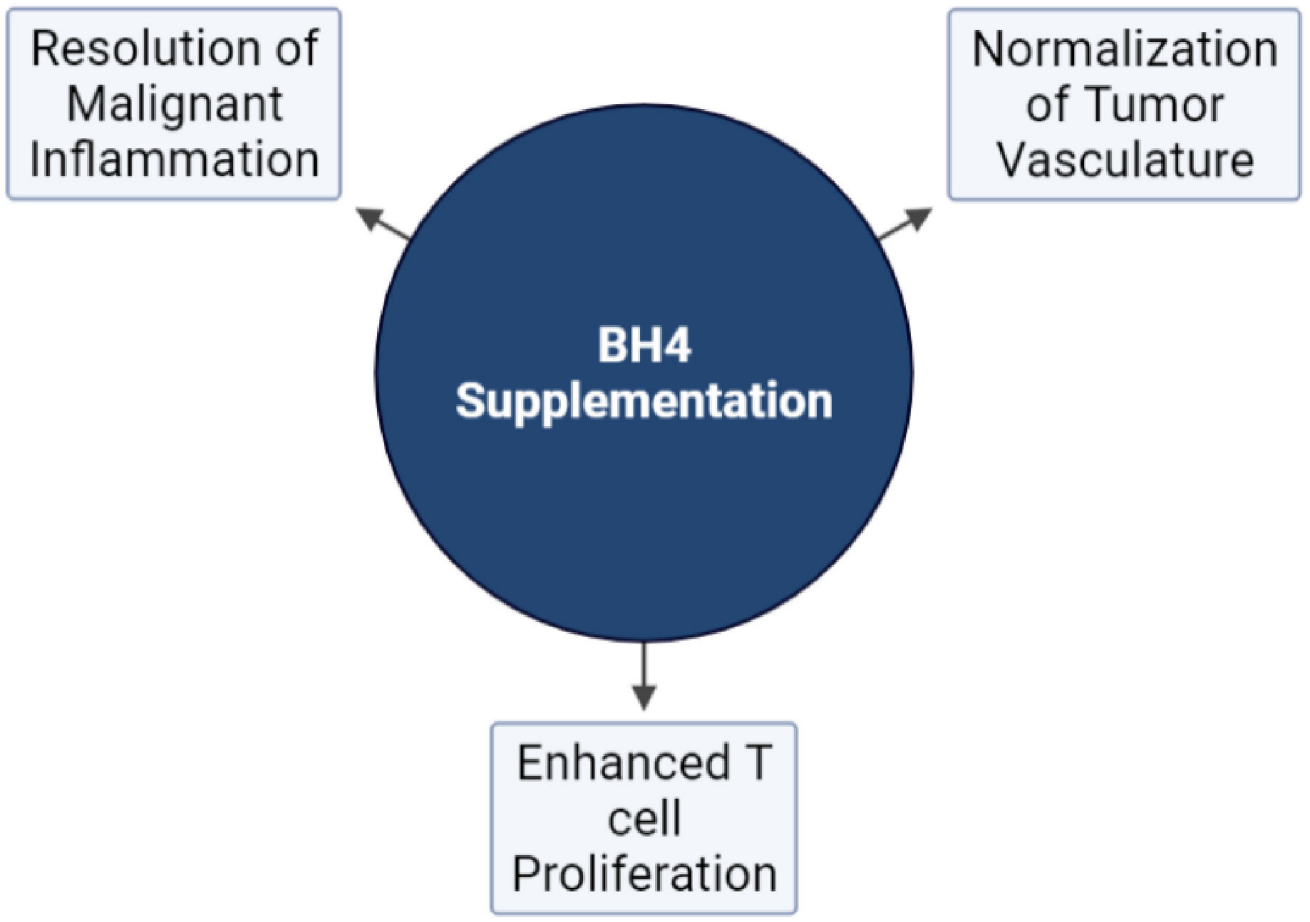
The effects of NOS recoupling on anti-tumor immunity. Exogenous BH4 supplementation has been demonstrated to affect three major hallmarks of tumorigenesis pertinent to the response to immunotherapy: The normalization of tumor vasculature, T cell proliferation, and the resolution of malignant inflammation.

Immune checkpoint blockade (ICB) has become the standard of care for multiple cancer types and members of this family of drugs have been approved for use as monotherapies for more than a dozen malignancies (101, 139, 140). However, compared to hematological malignancies (102), only a small fraction of patients with solid tumors respond to ICB monotherapy while as many as 87% of patients derive no clinical benefit (102, 141). It is believed that the TME stands as a major impediment to the effector function of CTLs, limiting the efficacy of therapies that rely on their activity. This has led to development of combination strategies designed to alter the TME, making tumors more susceptible to immune attack (123, 142–144). BH4 supplementation represent a novel approach to enhancing immunotherapy both by its direct proliferative effects on T cells outlined above and by its impact on multiple aspects of the TME including its effects on vascular normalization and the resolution of malignant inflammation (**Figure 4**) (92) As monotherapies, anti-angiogenic agents have also failed to yield clinical benefits, due in part to both the toxic effects that these agents have on normal tissues as well as the rapidity with which tumors develop resistance (102, 145). On the other hand, multiple anti-angiogenesis and immunotherapy combinations have achieved significant clinical results (146–151), demonstrating the potential of this treatment approach. Combination therapy has been shown to sensitize tumors to ICB by enhancing T cell penetration, supporting the formation of high endothelial venules, reducing T cell exhaustion, suppressing immunosuppressive cells types, and increasing the recruitment of activated antigen presenting cells (APCs) to the tumor (144). This illustrates the role that the dysfunctional tumor vasculature has on immune escape. However, combination with existing anti-angiogenic faces significant hurdles. For instance, the efficacy of combination relies on using strategies that normalize tumor vasculature and avoids detrimental vascular pruning as described above (27). That makes combination therapy a delicate balance, which may be difficult to achieve reliably in the clinic. In addition (144), most anti-angiogenic strategies are complicated by toxic sequelae due to their effects on normal tissues. This includes hypertension, proteinuria, impaired wound healing, and dose limiting cardiotoxicity (152–156). Finally, most anti-angiogenesis and vasculature normalizing strategies are only effective for a brief window of time (157). This is due to the rapid development of resistance to agents that target angiogenic pathways and represents a major obstacle to their clinical effectiveness.

For these reasons BH4 supplementation maybe be a more robust and reliable agent for combination therapy. Firstly (32, 158) the vascular normalizing function of sepiapterin does not rely on antagonizing angiogenic pathways but instead causes signaling within tumor vasculature to better resemble that of vasculature elsewhere in the body, as detailed above. Therefore, the risk of inducing vascular pruning as opposed to normalization is minimal and it likewise may not be as susceptible to the same mechanisms of therapeutic resistance as other vasculature normalizing strategies. Additionally, we have been able to demonstrate that enhanced tumor oxygenation caused by oral treatment with sepiapterin lasts for a significant amount of time after treatment has ceased (32), possibly reflecting its different mechanism of action. In addition, it demonstrates none of the toxic effects seen with other agents and has minimal detectable impact on normal tissues where BH4/BH2 levels are tightly regulated (158). This significantly widens the therapeutic window of BH4 supplementation compared to other agents.

Somewhat paradoxically, the quality of the unresolving, malignant inflammation typical of TME has long been recognized as a hallmark of tumorigenesis that aids in immune escape. As such, many strategies have been attempted to make tumors more susceptible to immunotherapy by limiting this inflammation (123, 159). Despite compelling pre-clinical reports, clinical trials aimed at inhibiting chronic inflammation to enhance therapeutic outcomes of immunotherapy have yet to yield significant results (123). Among other things, this may reflect the necessity of physiological inflammation for the function of immune cells in the TME. Similar to other anti-inflammatory/immunotherapy combination strategies, the resolution of tumor-supporting, malignant inflammation caused by uncoupled NOS may leave tumors vulnerable to more appropriate, T cell mediated responses. However, as with all such strategies timing and dosage need careful consideration, as immunotherapy relies on inflammation driven penetration and activation of both myeloid cells and lymphocytes into tumors. Interventions that limit myeloid cell activation in the TME or immune cell trafficking to the tumor tissue may limit immunotherapy when not given concomitantly with drivers of appropriate inflammation such as ICB or PRR agonists.

## Radiotherapy and the optimization of radio-immunotherapy

The majority of cancer patients with solid tumors will receive some form of radiotherapy (RT) during their course of treatment. However, treatment is limited by the toxic effects of off-target radiation on normal tissues. Much research in the field of radiation oncology is focused on limiting these off-target effects or enhancing the efficacy of RT in order to widen its therapeutic window. Numerous studies have demonstrated that NOS can be activated or upregulated by cancer cells in response to exposure to ionizing radiation (160, 161). However, it has also been demonstrated that oxidative stress caused by ionizing radiation may reduce BH4 availability resulting in a shift in BH4/BH2 ratios at both systemic and local levels (48, 162), resulting in uncoupled NOS activity (48). Suggested mechanisms for this phenomenon include the direct oxidation of BH4 by radiation-stimulated ROS production and the upregulation of the negative regulator of GCH1, GTP cyclohydrolase I feedback regulator (GFRP) (163, 164), although other pathways have been implicated (48). It is still unknown which pathway is dominant within the irradiated TME. As a result, multiple investigations are currently studying the role of NOS uncoupling in the response of tumors and normal tissues to RT as well as the therapeutic potential of BH4 supplementation.

The bulk of irradiated cancer cells die due to mitotic crisis, an ill-defined form of necrotic cell death caused by continued cell cycling in the presence of unrepaired DNA double-strand breaks (DSBs). These breaks are caused by either direct attack by high-energy photons or, much more commonly, by reactive oxygen (ROS) released from mitochondria within irradiated cells (165). Because the availability of ambient oxygen is necessary for the majority of DNA damage produced by radiation in the X-ray range, tumor hypoxia reduces the effectiveness of the radiation (157). Multiple studies have demonstrated that the degree of hypoxia directly correlates with reduced radiosensitivity by limiting the oxygen available for the production of ROS (166–169). Due to its ability to normalize tumor vasculature via NOS recoupling, BH4 supplementation has been demonstrated to enhance RT induced cell killing by increasing perfusion and oxygen delivery to tumor cells (32, 170). It is important to note that BH4 itself has been shown to act as a free radical scavenger and inhibitor of apoptosis *in vitro*, which may directly limit RT cell killing (48). In addition, both positive and negative effects of NO on apoptosis have been reported to function through various mechanisms *in vitro* as well (171). However, these may not be salient features of BH4 supplementation *in vivo* as they appear to be overshadowed by the positive effect generated by alleviating tumor hypoxia.

The total dose of RT given to a singular disease site is often limited due to the need to avoid lethal fibrotic side effects in soft tissues such as the heart and lungs. Radiation-induced cardiomyopathy is a dose-dependent, progressive disease characterized by myocardial and pericardial fibrosis, loss of contractile reserve and LV systolic function, and ultimately, premature death (172–175). Radiotherapy-induced lung fibrosis is a fibrotic disease resembling idiopathic pulmonary fibrosis and is one of the most common side effects of thoracic RT. Unfortunately, options for limiting radiation off-target effects or treating exposure are limited (48). The mechanisms behind radiation-induced tissue injury have been reviewed by other authors and will not be deeply examined here (176). However, multiple studies demonstrate that radiation-induced injury to the heart and lungs is mediated in part by chronic inflammatory signaling maintained by persistent endothelial dysfunction (175–181). Radiation-induced oxidative stress has been demonstrated to induce endothelial dysfunction by reducing BH4 levels leading to the uncoupling of NOS in both the heart and lungs (162, 163, 182). Multiple groups have reported that BH4 supplementation has a radioprotective effect on normal tissues through reversing the effects of uncoupling, restoring NO levels, mitigating inflammation, and acting as a free radical scavenger (48, 183). Three clinical trials are currently underway to study the effects of BH4 supplementation on radiation-induced skin injury and enteritis (NCT05299203, NCT05114226, NCT05138887). The results of these studies may open up a new avenue for the prevention of radiation-induced late effects and their life-threatening fibrotic sequelae, potentially allowing for more aggressive, sub-fractionated therapy without the fear of soft tissue fibrotic effects.

The response rates of solid tumors to immunotherapeutic strategies that have been effective for hematologic malignancies have been disappointing (142). It has been hypothesized that the cause of this discrepancy may lie in the ability of tumor-associated stroma to suppress the effector functions of anti-tumor lymphocytes. One proposed approach to overcoming this immunosuppressive barrier is combination with RT, which is thought to have multiple immunostimulatory effects (142, 165, 184–186). In brief, RT expands the repertoire of tumor-associated antigens (TAAs) available in the TME while directly stimulating type I IFN expression by activating cyclic-GAMP synthase (cGAS) and its downstream signaling partner stimulator of interferon genes (STING). This leads to the necessary cross-presentation of TAAs by newly recruited APCs and subsequent priming of tumor-specific CTLs. It is hoped that the addition of strategies that revitalize T cell function, such as ICB, may have synergistic effects to achieve tumor regression in patients with advanced disease. However, clinical trials exploring the efficacy of a radio-immunotherapy combination have so far failed to yield significant positive results.

**Figure 5:**
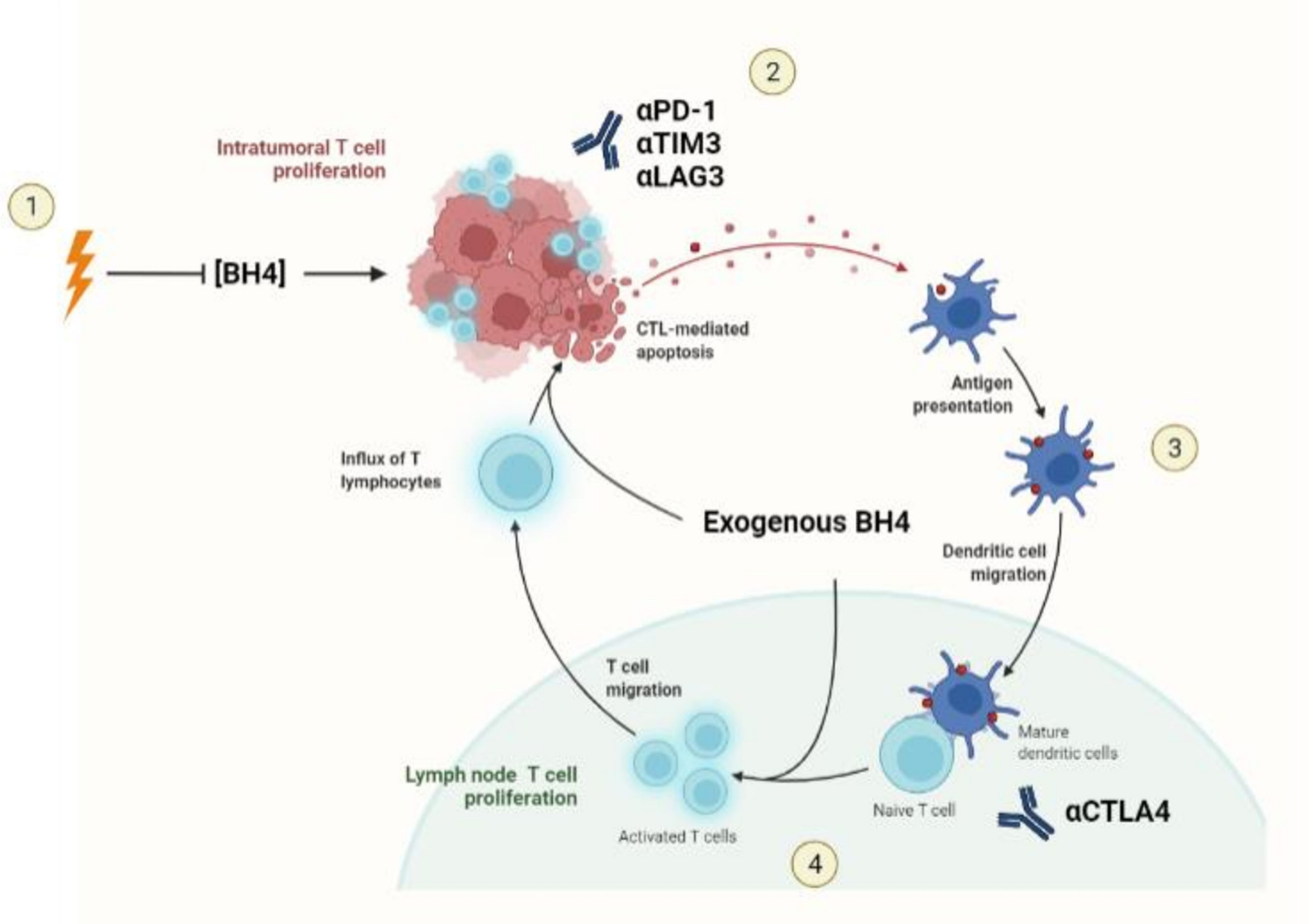
BH4 supplementation and Radiotherapy-Immunotherapy combination. 1) Radiotherapy stimulates an influx of lymphocytes and myeloid cells from the blood stream while simultaneously depleting BH4 in the TME. This depletion represents a potential barrier to ICB response in irradiated tumors. 2) Exogenous supplementation of BH4 leads to normalization of BH4 levels within the tumor, allowing for the intra-tumoral T cell proliferation necessary for the response to ICBs. 3) APCs recruited to the tumor acquire TAAs and migrate to the draining lymph nodes where they present antigen to naïve T cells. 4) Exogenous BH4 supplementation enhances tumor specific T cell expansion both enhancing the number of effector T cells available to attack the tumor as well as to be retained in the form of memory T cells.

Considering the negative effect that RT has on BH4 availability, RT-induced BH4 depletion may be an overlooked restraint to the immune response to irradiated tumors. BH4 supplementation may be exploited to potentiate the combination therapy for at meaningful clinical impact. First, systemic BH4 supplementation may enhance the proliferation of lymphocytes stimulated by APCs in the lymph nodes in response to RT (**Figure 5**). By enhancing the expansion of tumor-reactive lymphocytes, BH4 may provide for a more robust response to subsequent immune augmentation with ICB. Second, it has been established by multiple groups that durable anti-tumor immune responses to immunotherapy require intra-tumoral proliferation of T cells (187), a process that may be directly inhibited by RT-induced NOS uncoupling and local BH4 depletion. BH4 supplementation is likely to alleviate this immune-inhibitory process, leading to a more robust immune effector response within the irradiated TME.

RT also induces other changes to the TME that are relevant to the discussion of NOS uncoupling and BH4 metabolism. For instance, within a certain dose range, RT has a transient, type I IFN-dependent, normalizing effect on the tumor vasculature. It is thought that this may aid immune cell infiltration into the tumor and synergize with other vasculature normalizing strategies such as BH4 supplementation and immune checkpoint blockade (188). However, with so many vascular modulators at play, this three-part interaction will have to be carefully studied for potential synergy as it may tip the scales from immunostimulatory vascular normalization to immunosuppressive vascular regression and hypoxia. The immunostimulatory effects of RT have also been attributed its ability to recruit lymphocytes and myeloid cells. Many of these cells are likely to carry active iNOS. However, further study is required to understand how forced coupling of this influx of NOS by BH4 saturation may influence the irradiated TME. Finally, the advent of stereotactic body RT has made the immune-mediated effects of RT more clinically relevant by allowing the use of more immunogenic doses of radiation (8-15 Gy per fraction) (143). The radioprotective effect of BH4 supplementation on normal tissue may also aid in the use of these more immunogenic doses, leading to better synergy with immunotherapy. How these effects are incorporated into clinical practice will be exciting to see moving forward.

## Safety of BH4 supplementation

BH4 supplementation, either with BH4 itself or with its precursor sepiapterin, has a well-established safety record in humans. Both have undergone numerous clinical trials and appear to cause few side effects, even at very high levels (41–45). BH4 is currently indicated in the long-term setting for certain types of phenylketouria (189). This is contrary to other immunomodulators, such as pattern recognition agonists, which cause global immune activation and may have a narrow therapeutic window (190). This superior safety profile is likely a result of BH4s water solubility, its ease of excretion, its highly regulated concentrations in normal tissues (158), and the restricted nature of its positive effects on proliferating T cells (35). T cell proliferation and cytokine production are usually coupled upon activation. Interventions such as pegylated IFN-a, PRR agonism, or ICBs typically enhance both the number and activation state of T cells. However, these therapies enhance T cell activity indiscriminately, putting patients at risk for off target effects and severe reactions caused by T cell cytokine production. These risks pose a major obstacle to the augmentation of cancer immunity in clinical trials. Due to its purely proliferative effect, BH4 supplementation enhances T activity only in those T cells that have already been stimulated by their cognate antigen, without effecting global T cell activation and IFN-y secretion (20). This makes it a much safer immunological option.

However, therapy with ICBs does carry the risk of life threatening immune-related adverse events (irADs) (102). In addition, it has previously been reported in preclinical models that forced overproduction of BH4 exacerbates certain T cell mediated autoimmune disease in mice (20). While it is true that immunological side effects have not been reported with long term BH4 treatment alone, combination therapy with ICB should be considered carefully for the risk of exacerbating the established risk for irADs. However, these events are clinically manageable, and we expect that existing interventions for irADs, namely corticosteroid treatment and ceasing therapy with the offending agent, would be effective in mitigating significant risk for irADs. Finally, due to its anti-inflammatory effect on myeloid cells, which are necessary for anti-tumor immune responses, prolonged BH4 supplementation as a single agent runs the theoretical risk of suppressing anti-tumor immunity, potentially allowing for tumor advancement, and making distant peripheral sites more hospitable to metastases. Although this currently remains a theoretical risk, care should be taken in trials not to expose patients with documented tumors to prolonged BH4 treatment alone.

## Conclusions and future directions

**Figure 6:**
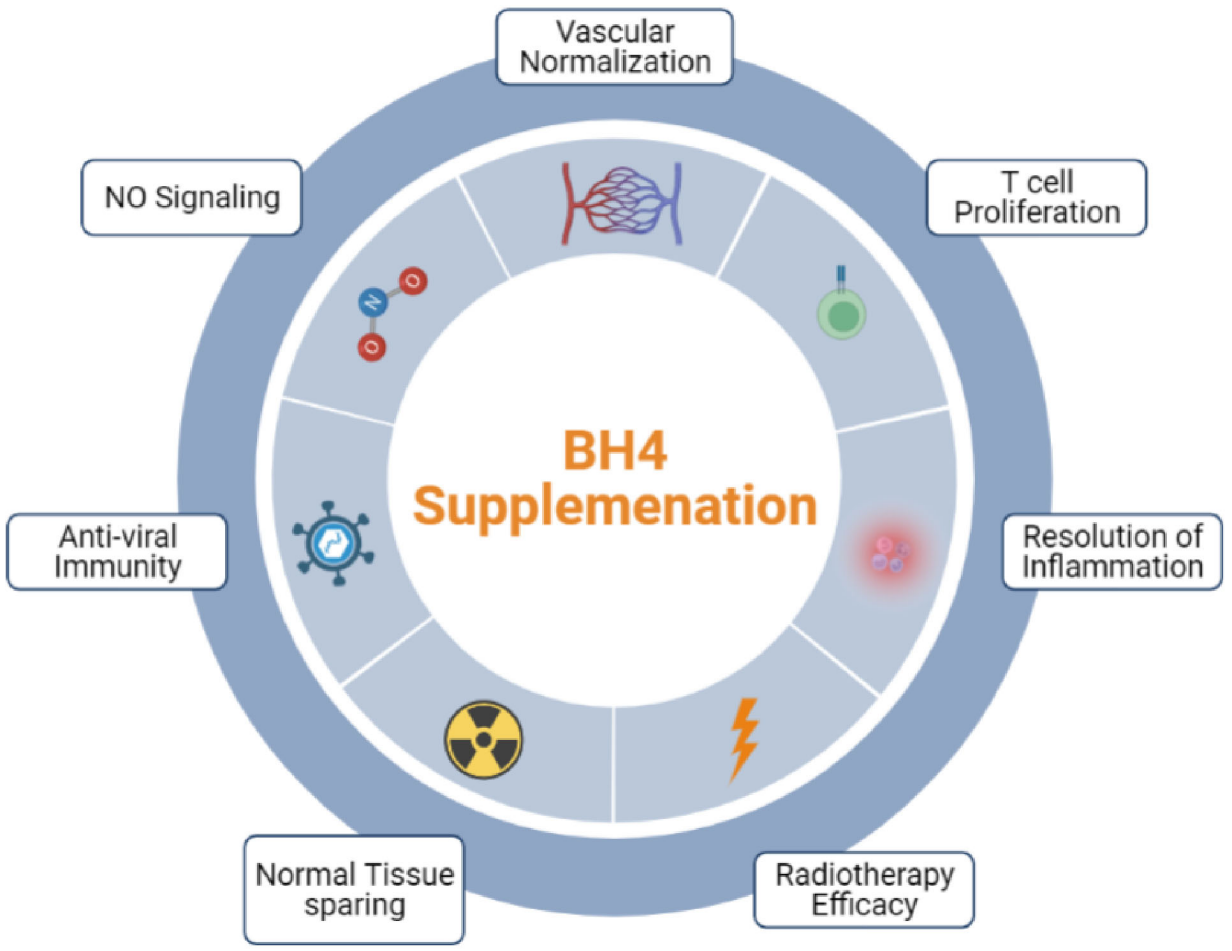
BH4 supplementation is poised to become a useful tool in the anti-cancer and anti-viral arsenal.

In conclusion, the propensity of low intratumoral BH4 levels to affect chronic inflammation, tumor-associated vascular dysfunction, and proliferation of CTLs contributes significantly to the tumor supportive characteristics of the TME. Here, we have provided pre-clinical evidence that supports exploring the clinical utility of BH4 supplementation to enhance existing anti-cancer therapies such as RT and immunotherapy. Whether BH4 restriction represents a bona fide, physiological mechanism of immune evasion or a pathological consequence of the disordered TME remains to be determined. Multiple strategies for enhancing BH4 production and recycling within cells exist beyond direct supplementation. One of these, supplementation with the BH4 precursor sepiapterin, is especially promising as it can be taken orally and can penetrate cells passively due to its relative hydrophobicity compared to BH4. This also has implications for tumors of the central nervous system as this form of biopterin can more easily pass through the blood-brain barrier (44). Therefore, BH4 supplementation may one day offer a new therapeutic avenue to oncologists and their patients (**Figure 6**).

## Author contributions

GC: Conceptualization, Writing – original draft, Writing – review & editing. AL: Writing – original draft, Writing – review & editing. AA: Writing – original draft, Writing – review & editing. ZL: Supervision, Writing – original draft, Writing – review & editing. X-YW: Supervision, Writing – original draft, Writing – review & editing.

## References

[1] Cai S Khoo J Channon KM. Augmented BH4 by gene transfer restores nitric oxide synthase function in hyperglycemic human endothelial cells. Cardiovasc Res (2005) 65:823–31. doi: 10.1016/j.cardiores.2004.10.040 15721862

[2] Wever RMF Van Dam T Van Rijn HJM De Groot F Rabelink TJ. Tetrahydrobiopterin regulates superoxide and nitric oxide generation by recombinant endothelial nitric oxide synthase. Biochem Biophys Res Commun (1997) 233:150–3. doi: 10.1006/bbrc.1997.7069 9268712

[3] Schmidt HHHW Hofmann H Schindler U Shutenko ZS Cunningham DD Feelisch M. No ·NO from NO synthase. Proc Natl Acad Sci USA (1996) 93:14492–7. doi: 10.1073/pnas.93.25.14492 PMC26160 8962079

[4] Cardnell RJG Rabender CS Ross GR Guo C Howlett EL Alam A. Sepiapterin ameliorates chemically induced murine colitis and azoxymethane-induced colon cancer. J Pharmacol Exp Ther (2013) 347:117–25. doi: 10.1124/jpet.113.203828 PMC3781406 23912334

[5] Rabender CS Alam A Sundaresan G Cardnell RJ Yakovlev VA Mukhopadhyay ND. The role of nitric oxide synthase uncoupling in tumor progression. Mol Cancer Res (2015) 13:1034–43. doi: 10.1158/1541-7786.MCR-15-0057-T PMC4470720 25724429

[6] Vásquez-Vivar J Kalyanaraman B Martásek P Hogg N Masters BSS Karoui H. Superoxide generation by endothelial nitric oxide synthase: The influence of cofactors. Proc Natl Acad Sci USA (1998) 95:9220–5. doi: 10.1073/pnas.95.16.9220 PMC21319 9689061

[7] Vśquez-Vivar J Kalyanaraman B Martásek P. The role of tetrahydrobiopterin in superoxide generation from eNOS: Enzymology and physiological implications. Free Radic Res (2003) 37:121–7. doi: 10.1080/1071576021000040655 12653200

[8] Stuehr D Pou S Rosen GM. Oxygen reduction by nitric-oxide synthases. J Biol Chem (2001) 276:14533–6. doi: 10.1074/jbc.R100011200 11279231

[9] Mikkelsen RB Wardman P. Biological chemistry of reactive oxygen and nitrogen and radiation-induced signal transduction mechanisms. Oncogene (2003) 22:5734–54. doi: 10.1038/sj.onc.1206663 12947383

[10] Alp NJ Channon KM. Regulation of endothelial nitric oxide synthase by tetrahydrobiopterin in vascular disease. Arterioscler Thromb Vasc Biol (2004) 24:413–20. doi: 10.1161/01.ATV.0000110785.96039.f6 14656731

[11] Alkaitis MS Crabtree MJ. Recoupling the cardiac nitric oxide synthases: Tetrahydrobiopterin synthesis and recycling. Curr Heart Fail Rep (2012) 9:200–10. doi: 10.1007/s11897-012-0097-5 PMC3406312 22711313

[12] Kuzkaya N Weissmann N Harrison DG Dikalov S. Interactions of peroxynitrite, tetrahydrobiopterin, ascorbic acid, and thiols: Implications for uncoupling endothelial nitric-oxide synthase. J Biol Chem (2003) 278:22546–54. doi: 10.1074/jbc.M302227200 12692136

[13] Werner-Felmayer G Golderer G Werner E. Tetrahydrobiopterin biosynthesis, utilization and pharmacological effects. Curr Drug Metab (2005) 3:159–73. doi: 10.2174/1389200024605073 12003348

[14] Wculek SK Cueto FJ Mujal AM Melero I Krummel MF Sancho D. Dendritic cells in cancer immunology and immunotherapy. Nat Rev Immunol (2020) 20:7–24. doi: 10.1038/s41577-019-0210-z 31467405

[15] Chow A Perica K Klebanoff CA Wolchok JD. Clinical implications of t cell exhaustion for cancer immunotherapy. Nat Rev Clin Oncol (2022) 19:775–90. doi: 10.1038/s41571-022-00689-z PMC10984554 36216928

[16] Zuazo M Arasanz H Bocanegra A Fernandez G Chocarro L Vera R. Systemic CD4 immunity as a key contributor to PD-L1/PD-1 blockade immunotherapy efficacy. Front Immunol (2020) 11:586907. doi: 10.3389/fimmu.2020.586907 33329566 PMC7734243

[17] Joffre OP Segura E Savina A Amigorena S. Cross-presentation by dendritic cells. Nat Rev Immunol (2012) 12:557–69. doi: 10.1038/nri3254 22790179

[18] Tay RE Richardson EK Toh HC. Revisiting the role of CD4+ t cells in cancer immunotherapy–new insights into old paradigms. Cancer Gene Ther (2021) 28:5–17. doi: 10.1038/s41417-020-0183-x 32457487 PMC7886651

[19] Raskov H Orhan A Christensen JP Gögenur I. Cytotoxic CD8+ t cells in cancer and cancer immunotherapy. Br J Cancer (2021) 124:359–67. doi: 10.1038/s41416-020-01048-4 PMC7853123 32929195

[20] Cronin SJF Seehus C Weidinger A Talbot S Reissig S Seifert M. The metabolite BH4 controls t cell proliferation in autoimmunity and cancer. Nature (2018). doi: 10.1038/s41586-018-0701-2 PMC6438708 30405245

[21] Bogdan C. Nitric oxide synthase in innate and adaptive immunity: An update. Trends Immunol (2015) 36:161–78. doi: 10.1016/j.it.2015.01.003 25687683

[22] Sailer S Keller MA Werner ER Watschinger K. The emerging physiological role of agmo 10 years after its gene identification. Life (2021) 11:88. doi: 10.3390/life11020088 33530536 PMC7911779

[23] Platten M Nollen EAA Röhrig UF Fallarino F Opitz CA. Tryptophan metabolism as a common therapeutic target in cancer, neurodegeneration and beyond. Nat Rev Drug Discovery (2019) 18:379–401. doi: 10.1038/s41573-019-0016-5 30760888

[24] Gargaro M Manni G Scalisi G Puccetti P Fallarino F. Tryptophan metabolites at the crossroad of immune-cell interaction *via* the aryl hydrocarbon receptor: Implications for tumor immunotherapy. Int J Mol Sci (2021) 22:4644. doi: 10.3390/ijms22094644 33924971 PMC8125364

[25] Benest AV Stone OA Miller WH Glover CP Uney JB Baker AH. Arteriolar genesis and angiogenesis induced by endothelial nitric oxide synthase overexpression results in a mature vasculature. Arterioscler Thromb Vasc Biol (2008) 28:1462–8. doi: 10.1161/ATVBAHA.108.169375 PMC2566940 18497305

[26] Sonveaux P Brouet A Havaux X Grégoire V Dessy C Balligand JL. Irradiation-induced angiogenesis through the up-regulation of the nitric oxide pathway: Implications for tumor radiotherapy. Cancer Res (2003) 63:1012–9. doi: 10.1016/s0167-8140(03)80572-8 12615716

[27] Rabender C Bruno N Wang L Zweit J Mikkelsen R Gobalakrishnan S. Optoacoustic imaging reveals normalized tumor oxygenation following sepiapterin treatment. J Nucl Med (2016) 57:1147.

[28] Mikkelsen RB Yakovlev VA Rabender CS Alam A. Nitric oxide synthase uncoupling in tumor progression and cancer therapy. Strategies to Enhance Ther Ratio Radiat as Cancer Treat (2016), 1034–43. doi: 10.1007/978-3-319-45594-5_6 PMC4470720 25724429

[29] Kashiwagi S Tsukada K Xu L Miyazaki J Kozin SV Tyrrell JA. Perivascular nitric oxide gradients normalize tumor vasculature. Nat Med (2008) 14:255–7. doi: 10.1038/nm1730 18278052

[30] Babykutty S Heishi T Tsukada K Huang Y Kozin S Conner D. Restoring perivascular nitric oxide gradients normalizes breast cancer vasculature. Nitric Oxide (2014) 42:115. doi: 10.1016/j.niox.2014.09.051

[31] Schaaf MB Garg AD Agostinis P. Defining the role of the tumor vasculature in antitumor immunity and immunotherapy article. Cell Death Dis (2018) 9 9(2):115. doi: 10.1038/s41419-017-0061-0 29371595 PMC5833710

[32] Rabender CS Bruno N Alam A Sundaresan G Zweit J Mikkelsen RB. Sepiapterin enhances tumor radio- and chemosensitivities by promoting vascular normalization. J Pharmacol Exp Ther (2018) 365:536–43. doi: 10.1124/jpet.117.245258 PMC11046730 29581154

[33] De Sanctis F Lamolinara A Boschi F Musiu C Caligola S Trovato R. Interrupting the nitrosative stress fuels tumor-specific cytotoxic t lymphocytes in pancreatic cancer. J Immunother Cancer (2022) 10:e003549. doi: 10.1136/jitc-2021-003549 35022194 PMC8756272

[34] Cronin SJF Penninger JM. From t-cell activation signals to signaling control of anti-cancer immunity. Immunol Rev (2007) 220:151–68. doi: 10.1111/j.1600-065X.2007.00570.x 17979845

[35] Cronin SJF Woolf CJ Weiss G Penninger JM. The role of iron regulation in immunometabolism and immune-related disease. Front Mol Biosci (2019) 22:116. doi: 10.3389/fmolb.2019.00116 PMC6883604 31824960

[36] Wang S Xu J Song P Wu Y Zhang J Choi HC. Acute inhibition of guanosine triphosphate cyclohydrolase 1 uncouples endothelial nitric oxide synthase and elevates blood pressure. Hypertension (2008) 52:484–90. doi: 10.1161/HYPERTENSIONAHA.108.112094 PMC3523107 18645049

[37] Roe ND He EY Wu Z Ren J. Folic acid reverses nitric oxide synthase uncoupling and prevents cardiac dysfunction in insulin resistance: Role of Ca2+/calmodulin-activated protein kinase II. Free Radic Biol Med (2013) 65:234–43. doi: 10.1016/j.freeradbiomed.2013.06.042 PMC3859865 23820268

[38] Shimazu T Otani H Yoshioka K Fujita M Okazaki T Iwasaka T. Sepiapterin enhances angiogenesis and functional recovery in mice after myocardial infarction. Am J Physiol Heart Circ Physiol (2011) 301:H2061–72. doi: 10.1152/ajpheart.00525.2011 21890687

[39] Jo H Otani H Jo F Shimazu T Okazaki T Yoshioka K. Inhibition of nitric oxide synthase uncoupling by sepiapterin improves left ventricular function in streptozotocin-induced diabetic mice. Clin Exp Pharmacol Physiol (2011) 38:485–93. doi: 10.1111/j.1440-1681.2011.05535.x 21554376

[40] Carnicer R Duglan D Ziberna K Recalde A Reilly S Simon JN. BH4 increases nNOS activity and preserves left ventricular function in diabetes. Circ Res (2021) 128:585–601. doi: 10.1161/CIRCRESAHA.120.316656 33494625 PMC7612785

[41] Abell T Garcia LM Wiener G Wo JM Bulat RS Smith N. Su1719 IMPROVED GASTRIC ACCOMMODATION IN WOMEN WITH MODERATE OR SEVERE DIABETIC GASTROPARESIS RANDOMIZED TO ORAL CNSA-001 (SEPIAPTERIN) VERSUS PLACEBO. Gastroenterology (2020) 158:S1–S1603. doi: 10.1016/s0016-5085(20)32247-2

[42] Abell TL Garcia LM Wiener GJ Wo JM Bulat RS Smith N. Effect of oral CNSA-001 (sepiapterin, PTC923) on gastric accommodation in women with diabetic gastroparesis: A randomized, placebo-controlled, phase 2 trial. J Diabetes Complications (2021) 35. doi: 10.1016/j.jdiacomp.2021.107961 34176722

[43] Bratkovic D Margvelashvili L Tchan MC Nisbet J Smith N. PTC923 (sepiapterin) lowers elevated blood phenylalanine in subjects with phenylketonuria: a phase 2 randomized, multi-center, three-period crossover, open-label, active controlled, all-comers study. Metabolism (2022) 128:155116. doi: 10.1016/j.metabol.2021.155116 34973284

[44] Smith N Longo N Levert K Hyland K Blau N. Exploratory study of the effect of one week of orally administered CNSA-001 (sepiapterin) on CNS levels of tetrahydrobiopterin, dihydrobiopterin and monoamine neurotransmitter metabolites in healthy volunteers. Mol Genet Metab Rep (2019) 21:100500. doi: 10.1016/j.ymgmr.2019.100500 31453106 PMC6700519

[45] Smith N Longo N Levert K Hyland K Blau N. Phase i clinical evaluation of CNSA-001 (sepiapterin), a novel pharmacological treatment for phenylketonuria and tetrahydrobiopterin deficiencies, in healthy volunteers. Mol Genet Metab (2019) 126:406–12. doi: 10.1016/j.ymgme.2019.02.001 30922814

[46] Panieri E Santoro MM. Ros homeostasis and metabolism: A dangerous liason in cancer cells. Cell Death Dis (2016) 7:e2253. doi: 10.1038/cddis.2016.105 27277675 PMC5143371

[47] Crabtree MJ Smith CL Lam G Goligorsky MS Gross SS. Ratio of 5,6,7,8-tetrahydrobiopterin to 7,8-dihydrobiopterin in endothelial cells determines glucose-elicited changes in NO vs. superoxide production by eNOS. Am J Physiol Heart Circ Physiol (2008) 294:H1530–40. doi: 10.1152/ajpheart.00823.2007 PMC2722919 18192221

[48] Feng Y Feng Y Gu L Liu P Cao J Zhang S. The critical role of tetrahydrobiopterin (BH4) metabolism in modulating radiosensitivity: BH4/NOS axis as an angel or a devil. Front Oncol (2021) 11:720632. doi: 10.3389/fonc.2021.720632 34513700 PMC8429800

[49] Zheng JS Yang XQ Lookingland KJ Fink GD Hesslinger C Kapatos G. Gene transfer of human guanosine 5′-triphosphate cyclohydrolase i restores vascular tetrahydrobiopterin level and endothelial function in low renin hypertension. Circulation (2003) 108:1238–45. doi: 10.1161/01.CIR.0000089082.40285.C3 12925450

[50] Mitchell BM Dorrance AM Webb RC. GTP cyclohydrolase 1 downregulation contributes to glucocorticoid hypertension in rats. Hypertension (2003) 41:669–74. doi: 10.1161/01.HYP.0000051889.62249.5D 12623977

[51] Fanet H Capuron L Castanon N Calon F Vancassel S. Tetrahydrobioterin (BH4) pathway: From metabolism to neuropsychiatry. Curr Neuropharmacol (2020) 19:591–609. doi: 10.2174/1570159x18666200729103529 PMC8573752 32744952

[52] Nathan JA. Nitrosylation rewires metabolism. Nat Chem Biol (2023) 19:253–4. doi: 10.1038/s41589-022-01169-2 36266350

[53] Zhou HL Zhang R Anand P Stomberski CT Qian Z Hausladen A. Metabolic reprogramming by the s-nitroso-CoA reductase system protects against kidney injury. Nature (2019) 565:96–100. doi: 10.1038/s41586-018-0749-z 30487609 PMC6318002

[54] Seth D Hess DT Hausladen A Wang L Juan WY Stamler JS. A multiplex enzymatic machinery for cellular protein s-nitrosylation. Mol Cell (2018). doi: 10.1016/j.molcel.2017.12.025 PMC5999318 29358078

[55] Stomberski CT Hess DT Stamler JS. Protein s-nitrosylation: Determinants of specificity and enzymatic regulation of s-Nitrosothiol-Based signaling. Antioxid Redox Signal (2019) 30:1331–51. doi: 10.1089/ars.2017.7403 PMC6391618 29130312

[56] Jia J Arif A Terenzi F Willard B Plow EF Hazen SL. Target-selective protein s-nitrosylation by sequence motif recognition. Cell (2014) 59:623–34. doi: 10.1016/j.cell.2014.09.032 PMC4243042 25417112

[57] Wink DA Mitchell JB. Chemical biology of nitric oxide: Insights into regulatory, cytotoxic, and cytoprotective mechanisms of nitric oxide. Free Radic Biol Med (1998) 25:434–56. doi: 10.1016/S0891-5849(98)00092-6 9741580

[58] Jourd’heuil D Miranda KM Kim SM Espey MG Vodovotz Y Laroux S. The oxidative and nitrosative chemistry of the nitric oxide/superoxide reaction in the presence of bicarbonate. Arch Biochem Biophys (1999) 365:92–100. doi: 10.1006/abbi.1999.1143 10222043

[59] Kelleher ZT Matsumoto A Stamler JS Marshall HE. NOS2 regulation of NF-κB by s-nitrosylation of p65. J Biol Chem (2007) 282:30667–72. doi: 10.1074/jbc.M705929200 17720813

[60] Marshall HE Hess DT Stamler JS. S-nitrosylation: Physiological regulation of NF-κB. Proc Natl Acad Sci USA (2004) 101:8841–2. doi: 10.1073/pnas.0403034101 PMC428432 15187230

[61] Marshall HE Stamler JS. Inhibition of NF-κB by s-nitrosylation. Biochemistry (2001) 40:1688–93. doi: 10.1021/bi002239y 11327828

[62] Bayden AS Yakovlev VA Graves PR Mikkelsen RB Kellogg GE. Factors influencing protein tyrosine nitration-structure-based predictive models. Free Radic Biol Med (2011) 50:749–62. doi: 10.1016/j.freeradbiomed.2010.12.016 PMC3039091 21172423

[63] Yakovlev VA Barani IJ Rabender CS Black SM Leach JK Graves PR. Tyrosine nitration of IκBα: A novel mechanism for NF-κB activation. Biochemistry (2007) 46:11671–83. 10.1021/bi701107z PMC2678910 17910475

[64] Schmitz K Trautmann S Hahnefeld L Fischer C Schreiber Y Wilken-Schmitz A. Sapropterin (BH4) aggravates autoimmune encephalomyelitis in mice. Neurotherapeutics (2021) 18:1862–79. doi: 10.1007/s13311-021-01043-4 PMC8609075 33844153

[65] Koh KP Wang Y Yi T Shiao SL Lorber MI Sessa WC. T cell-mediated vascular dysfunction of human allografts results from IFN-γ dysregulation of NO synthase. J Clin Invest (2004) 114:846–56. doi: 10.1172/JCI21767 PMC516264 15372109

[66] Choy JC Wang Y Tellides G Pober JS. Induction of inducible NO synthase in bystander human t cells increases allogenic response in the vasculature. Proc Natl Acad Sci USA (2007) 104:1313–8. doi: 10.1073/pnas.0607731104 PMC1783095 17227851

[67] Nagy G Koncz A Perl A. T cell activation-induced mitochondrial hyperpolarization is mediated by ca 2+ - and redox-dependent production of nitric oxide. J Immunol (2003) 171:5188–97. doi: 10.4049/jimmunol.171.10.5188 PMC4078644 14607919

[68] Reiling N Kröncke R Ulmer AJ Gerdes J Flad HD Hauschildt S. Nitric oxide synthese: Expression of the endothelial, Ca2+/calmodulin-dependent isoform in human b and t lymphocytes. Eur J Immunol (1996). doi: 10.1002/eji.1830260302 8605914

[69] Sciorati C Rovere P Ferrarini M Heltai S Manfredi AA Clementi E. Autocrine nitric oxide modulates CD95-induced apoptosis in γδ t lymphocytes. J Biol Chem (1997) 272:23211–5. doi: 10.1074/jbc.272.37.23211 9287328

[70] Ibiza S Víctor VM Boscá I Ortega A Urzainqui A O’Connor JE. Endothelial nitric oxide synthase regulates t cell receptor signaling at the immunological synapse. Immunity (2006) 24:753–65. doi: 10.1016/j.immuni.2006.04.006 16782031

[71] Ibiza S Pérez-Rodríguez A Ortega Á Martínez-Ruiz A Barreiro O García-Domínguez CA. Endothelial nitric oxide synthase regulates n-ras activation on the golgi complex of antigen-stimulated t cells. Proc Natl Acad Sci USA (2008) 105:10507–12. doi: 10.1073/pnas.0711062105 PMC2492470 18641128

[72] Sriskandan S Evans TJ Cohen J. Bacterial superantigen-induced human lymphocyte responses are nitric oxide dependent and mediated by IL-12 and IFN-γ. J Immunol (1996) 156(7):2430–5. 8786301

[73] Niedbala W Qing WX Campbell C Thomson D Komai-Koma M Liew FY. Nitric oxide preferentially induces type 1 t cell differentiation by selectively up-regulating IL-12 receptor β2 expression *via* cGMP. Proc Natl Acad Sci USA (2002) 99:16186–91. doi: 10.1073/pnas.252464599 PMC138586 12451176

[74] King MR Ismail AS Davis LS Karp DR. Oxidative stress promotes polarization of human t cell differentiation toward a t helper 2 phenotype. J Immunol (2006) 176:2765–72. doi: 10.4049/jimmunol.176.5.2765 16493032

[75] Chen W Li L Brod T Saeed O Thabet S Jansen T. Role of increased guanosine triphosphate cyclohydrolase-1 expression and tetrahydrobiopterin levels upon t cell activation. J Biol Chem (2011) 286:13846–51. doi: 10.1074/jbc.M110.191023 PMC3077585 21343293

[76] Lee S-W Choi H Eun S-Y Fukuyama S Croft M. Nitric oxide modulates TGF-β–directive signals to suppress Foxp3 + regulatory t cell differentiation and potentiate Th1 development. J Immunol (2011) 186:6972–80. doi: 10.4049/jimmunol.1100485 PMC3113707 21555530

[77] Brahmachari S Pahan K. Myelin basic protein priming reduces the expression of Foxp3 in t cells *via* nitric oxide. J Immunol (2010) 184:1799–809. doi: 10.4049/jimmunol.0804394 PMC2855656 20083653

[78] Chen CA Wang TY Varadharaj S Reyes LA Hemann C Talukder MAH. S-glutathionylation uncouples eNOS and regulates its cellular and vascular function. Nature (2010) 468:1115–8. doi: 10.1038/nature09599 PMC3370391 21179168

[79] O’Neill LAJ Kishton RJ Rathmell J. A guide to immunometabolism for immunologists. Nat Rev Immunol (2016) 16:553–65. doi: 10.1038/nri.2016.70 PMC5001910 27396447

[80] Haruki H Hovius R Pedersen MG Johnsson K. Tetrahydrobiopterin biosynthesis as a potential target of the kynurenine pathway metabolite xanthurenic acid. J Biol Chem (2016) 291:652–7. doi: 10.1074/jbc.C115.680488 PMC4705385 26565027

[81] Lu T Ramakrishnan R Altiok S Youn JI Cheng P Celis E. Tumor-infiltrating myeloid cells induce tumor cell resistance to cytotoxic t cells in mice. J Clin Invest (2011) 121:4015–29. doi: 10.1172/JCI45862 PMC3195459 21911941

[82] Lu T Gabrilovich DI. Molecular pathways: Tumor-infiltrating myeloid cells and reactive oxygen species in regulation of tumor microenvironment. Clin Cancer Res (2012) 291:652–7. doi: 10.1158/1078-0432.CCR-11-2939 PMC3445728 22718858

[83] Nagaraj S Gupta K Pisarev V Kinarsky L Sherman S Kang L. Altered recognition of antigen is a mechanism of CD8+ t cell tolerance in cancer. Nat Med (2007) 13:828–35. doi: 10.1038/nm1609 PMC2135607 17603493

[84] Molon B Ugel S Del Pozzo F Soldani C Zilio S Avella D. Chemokine nitration prevents intratumoral infiltration of antigen-specific t cells. J Exp Med (2011) 208:1949–62. doi: 10.1084/jem.20101956 PMC3182051 21930770

[85] Sato E Simpson KL Grisham MB Koyama S Robbins RA. Effects of reactive oxygen and nitrogen metabolites on RANTES- and IL- 5-induced eosinophil chemotactic activity in vitro. Am J Pathol (1999) 155:591–8. doi: 10.1016/S0002-9440(10)65154-1 PMC1866862 10433951

[86] Sato E Simpson KL Grisham MB Koyama S Robbins RA. Effects of reactive oxygen and nitrogen metabolites on MCP-1-induced monocyte chemotactic activity in vitro. Am J Physiol Lung Cell Mol Physiol (1999) 277:L543–9. doi: 10.1152/ajplung.1999.277.3.l543 10484461

[87] Cunha PP Bargiela D Minogue E Krause LCM Barbieri L Brombach C. Infiltration of tumors is regulated by t cell-intrinsic nitric oxide synthesis. Cancer Immunol Res (2023) 11:351–63. doi: 10.1158/2326-6066.CIR-22-0387 PMC9975666 36574610

[88] Fiorucci S Santucci L Antonelli E Distrutti E Del Sero G Morelli O. NO-aspirin protects from t cell-mediated liver injury by inhibiting caspase-dependent processing of Th1-like cytokines. Gastroenterology (2000) 18:404–21. doi: 10.1016/S0016-5085(00)70223-X 10648469

[89] Fiorucci S Mencarelli A Palazzetti B Del Soldato P Morelli A Ignarro LJ. An NO derivative of ursodeoxycholic acid protects against fas-mediated liver injury by inhibiting caspase activity. Proc Natl Acad Sci USA (2001) 98:2652–7. doi: 10.1073/pnas.041603898 PMC30193 11226294

[90] Huang FP Niedbala W Wei XQ Xu D Feng GJ Robinson JH. Nitric oxide regulates Th1 cell development through the inhibition of IL-12 synthesis by macrophages. Eur J Immunol (1998) 28:4062–70. doi: 10.1002/(SICI)1521-4141(199812)28:12<4062::AID-IMMU4062>3.0.CO;2-K 9862342

[91] Niedbala W Cai B Liu H Pitman N Chang L Liew FY. Nitric oxide induces CD4+CD25+ Foxp3- regulatory t cells from CD4+CD25- t cells *via* p53, IL-2, and OX40. Proc Natl Acad Sci USA (2007) 104:15478–83. doi: 10.1073/pnas.0703725104 PMC1978217 17875988

[92] Liu Z Wang Y Huang Y Kim BYS Shan H Wu D. Tumor vasculatures: A new target for cancer immunotherapy. Trends Pharmacol Sci (2019) 40:613–23. doi: 10.1016/j.tips.2019.07.001 PMC7925217 31331639

[93] Lee WS Yang H Chon HJ Kim C. Combination of anti-angiogenic therapy and immune checkpoint blockade normalizes vascular-immune crosstalk to potentiate cancer immunity. Exp Mol Med (2020) 52:1475–85. doi: 10.1038/s12276-020-00500-y PMC8080646 32913278

[94] Huang Y Yuan J Righi E Kamoun WS Ancukiewicz M Nezivar J. Vascular normalizing doses of antiangiogenic treatment reprogram the immunosuppressive tumor microenvironment and enhance immunotherapy. Proc Natl Acad Sci USA (2012). doi: 10.1073/pnas.1215397109 PMC3491458 23045683

[95] Stylianopoulos T Munn LL Jain RK. Reengineering the physical microenvironment of tumors to improve drug delivery and efficacy: From mathematical modeling to bench to bedside. Trends Cancer (2018) 4:292–319. doi: 10.1016/j.trecan.2018.02.005 29606314 PMC5930008

[96] Mpekris F Voutouri C Baish JW Duda DG Munn LL Stylianopoulos T. Combining microenvironment normalization strategies to improve cancer immunotherapy. Proc Natl Acad Sci USA (2020) 117:3728–37. doi: 10.1073/pnas.1919764117 PMC7035612 32015113

[97] Jain RK. Normalizing tumor microenvironment to treat cancer: Bench to bedside to biomarkers. J Clin Oncol (2013) 31:2205–18. doi: 10.1200/JCO.2012.46.3653 PMC3731977 23669226

[98] Huang Y Goel S Duda DG Fukumura D Jain RK. Vascular normalization as an emerging strategy to enhance cancer immunotherapy. Cancer Res (2013) 73:2943–8. doi: 10.1158/0008-5472.CAN-12-4354 PMC3655127 23440426

[99] Salmon H Franciszkiewicz K Damotte D Dieu-Nosjean MC Validire P Trautmann A. Matrix architecture defines the preferential localization and migration of t cells into the stroma of human lung tumors. J Clin Invest (2012) 122:899–910. doi: 10.1172/JCI45817 22293174 PMC3287213

[100] Mariathasan S Turley SJ Nickles D Castiglioni A Yuen K Wang Y. TGFβ attenuates tumour response to PD-L1 blockade by contributing to exclusion of t cells. Nature (2018) 554:544–8. doi: 10.1038/nature25501 PMC6028240 29443960

[101] Munn LL Jain RK. Vascular regulation of antitumor immunity. Sci (1979) (2019) 365:544–5. doi: 10.1126/science.aaw7875 PMC7321824 31395771

[102] Datta M Coussens LM Nishikawa H Hodi FS Jain RK. Reprogramming the tumor microenvironment to improve immunotherapy: Emerging strategies and combination therapies. Am Soc Clin Oncol Educ Book (2019) 39:165–74. doi: 10.1200/edbk_237987 PMC6596289 31099649

[103] Martin JD Cabral H Stylianopoulos T Jain RK. Improving cancer immunotherapy using nanomedicines: progress, opportunities and challenges. Nat Rev Clin Oncol (2020) 17:251–66. doi: 10.1038/s41571-019-0308-z PMC8272676 32034288

[104] Jain RK Stylianopoulos T. Delivering nanomedicine to solid tumors. Nat Rev Clin Oncol (2010) 7:653–64. doi: 10.1038/nrclinonc.2010.139 PMC3065247 20838415

[105] Noman MZ Desantis G Janji B Hasmim M Karray S Dessen P. PD-L1 is a novel direct target of HIF-1α, and its blockade under hypoxia enhanced MDSC-mediated t cell activation. J Exp Med (2014) 211:781–90. doi: 10.1084/jem.20131916 PMC4010891 24778419

[106] Noman MZ Hasmim M Messai Y Terry S Kieda C Janji B. Hypoxia: A key player in antitumor immune response. a review in the theme: Cellular responses to hypoxia. Am J Physiol Cell Physiol (2015) 309:C569–79. doi: 10.1152/ajpcell.00207.2015 PMC4628936 26310815

[107] Facciabene A Peng X Hagemann IS Balint K Barchetti A Wang LP. Tumour hypoxia promotes tolerance and angiogenesis *via* CCL28 and t reg cells. Nature (2011) 475:226–30. doi: 10.1038/nature10169 21753853

[108] Calcinotto A Filipazzi P Grioni M Iero M De Milito A Ricupito A. Modulation of microenvironment acidity reverses anergy in human and murine tumor-infiltrating t lymphocytes. Cancer Res (2012) 72:2746–56. doi: 10.1158/0008-5472.CAN-11-1272 22593198

[109] Palazón A Aragonés J Morales-Kastresana A Ortiz De Landázuri M Melero I. Molecular pathways: Hypoxia response in immune cells fighting or promoting cancer. Clin Cancer Res (2012) 18:1207–13. doi: 10.1158/1078-0432.CCR-11-1591 22205687

[110] Sonveaux P Frérart F Bouzin C Brouet A DeWever J Jordan BF. Irradiation promotes akt-targeting therapeutic gene delivery to the tumor vasculature. Int J Radiat Oncol Biol Phys (2007) 67:1155–62. doi: 10.1016/j.ijrobp.2006.11.031 17276618

[111] Falcón BL Hashizume H Koumoutsakos P Chou J Bready JV Coxon A. Contrasting actions of selective inhibitors of angiopoietin-1 and angiopoietin-2 on the normalization of tumor blood vessels. Am J Pathol (2009) 175:2159–70. doi: 10.2353/ajpath.2009.090391 PMC2774078 19815705

[112] Zacharek A Chen J Zhang C Cui X Roberts C Jiang H. Nitric oxide regulates Angiopoietin1/Tie2 expression after stroke. Neurosci Lett (2006) 404:28–32. doi: 10.1016/j.neulet.2006.05.027 16762501 PMC2791334

[113] Kobayashi H DeBusk LM Babichev YO Dumont DJ Lin PC. Hepatocyte growth factor mediates angiopoietin-induced smooth muscle cell recruitment. Blood (2006) 108:1260–6. doi: 10.1182/blood-2005-09-012807 PMC1895873 16638932

[114] Schulz E Jansen T Wenzel P Daiber A Münzel T. Nitric oxide, tetrahydrobiopterin, oxidative stress, and endothelial dysfunction in hypertension. Antioxid Redox Signal (2008) 10:1115–26. doi: 10.1089/ars.2007.1989 18321209

[115] Roe ND Ren J. Nitric oxide synthase uncoupling: A therapeutic target in cardiovascular diseases. Vascul Pharmacol (2012) 57:168–72. doi: 10.1016/j.vph.2012.02.004 22361333

[116] Fukumura D Kashiwagi S Jain RK. The role of nitric oxide in tumour progression. Nat Rev Cancer (2006) 6:521–34. doi: 10.1038/nrc1910 16794635

[117] Kashiwagi S Izumi Y Gohongi T Demou ZN Xu L Huang PL. NO mediates mural cell recruitment and vessel morphogenesis in murine melanomas and tissue-engineered blood vessels. J Clin Invest (2005) 115:1816–27. doi: 10.1172/JCI24015 PMC1143589 15951843

[118] Schleicher M Yu J Murata T Derakhshan B Atochin D Qian L. The Akt1-eNOS axis illustrates the specificity of kinase-substrate relationships in vivo. Sci Signal (2009) 2:ra41. doi: 10.1126/scisignal.2000343 19654415 PMC4750881

[119] Cardnell RJG Mikkelsen RB. Nitric oxide synthase inhibition enhances the antitumor effect of radiation in the treatment of squamous carcinoma xenografts. PloS One (2011) 6:e20147. doi: 10.1371/journal.pone.0020147 21647438 PMC3102067

[120] Ng QS Goh V Milner J Stratford MR Folkes LK Tozer GM. Effect of nitric-oxide synthesis on tumour blood volume and vascular activity: a phase i study. Lancet Oncol (2007) 8:111–8. doi: 10.1016/S1470-2045(07)70001-3 17267325

[121] Siemann DW. The unique characteristics of tumor vasculature and preclinical evidence for its selective disruption by tumor-vascular disrupting agents. Cancer Treat Rev (2011) 37:63–74. doi: 10.1016/j.ctrv.2010.05.001 20570444 PMC2958232

[122] Hanahan D Weinberg RA. The hallmarks of cancer review douglas. Cell (2000) 100:57–70. doi: 10.1016/s0092-8674(00)81683-9 10647931

[123] Crusz SM Balkwill FR. Inflammation and cancer: Advances and new agents. Nat Rev Clin Oncol (2015) 12:584–96. doi: 10.1038/nrclinonc.2015.105 26122183

[124] Kundu JK Surh YJ. Inflammation: Gearing the journey to cancer. Mutat Res Rev Mutat Res (2008) 659:15–30. doi: 10.1016/j.mrrev.2008.03.002 18485806

[125] Reynaert NL Ckless K Korn SH Vos N Guala AS Wouters EFM. Nitric oxide represses inhibitory κB kinase through s-nitrosylation. Proc Natl Acad Sci USA (2004) 101:8945–50. doi: 10.1073/pnas.0400588101 PMC428452 15184672

[126] Yakovlev VA Barani IJ Rabender CS Black SM Leach JK Graves PR. Tyrosine nitration of IκBα: A novel mechanism for NF-κB activation. Biochemistry (2007) 46:11671–83. doi: 10.1021/bi701107z PMC2678910 17910475

[127] Godoy LC Moretti AI Jurado MC Oxer D Janiszewski M Ckless K. Loss of CD40 endogenous s-nitrosylation during inflammatory response in endotoxemic mice and patients with sepsis. Shock (2010) 33:626–33. doi: 10.1097/SHK.0b013e3181cb88e6 20473113

[128] Lim SY Raftery M Cai H Hsu K Yan WX Hseih H-L. S -nitrosylated S100A8: Novel anti-inflammatory properties. J Immunol (2008) 181:5627–36. doi: 10.4049/jimmunol.181.8.5627 18832721

[129] Park HS Huh SH Kim MS Lee SH Choi EJ. Nitric oxide negatively regulates c-jun n-terminal kinase/stress-activated protein kinase by means of s-nitrosylation. Proc Natl Acad Sci USA (2000) 97:14382–7. doi: 10.1073/pnas.97.26.14382 PMC18927 11121042

[130] Park HS Mo JS Choi EJ. Nitric oxide inhibits an interaction between JNK1 and c-jun through nitrosylation. Biochem Biophys Res Commun (2006) 351:281–6. doi: 10.1016/j.bbrc.2006.10.034 17054907

[131] Smith BC Fernhoff NB Marletta MA. Mechanism and kinetics of inducible nitric oxide synthase auto- s -nitrosation and inactivation. Biochemistry (2012) 51:1028–40. doi: 10.1021/bi201818c PMC3277664 22242685

[132] Mitchell DA Erwin PA Michel T Marletta MA. S-nitrosation and regulation of inducible nitric oxide synthase. Biochemistry (2005) 44:4636–47. doi: 10.1021/bi0474463 15779890

[133] Lefer DJ Jones SP Girod WG Baines A Grisham MB Cockrell AS. Leukocyte-endothelial cell interactions in nitric oxide synthase- deficient mice. Am J Physiol Heart Circ Physiol (1999) 276:H1943–50. doi: 10.1152/ajpheart.1999.276.6.h1943 10362674

[134] Sanz MJ Hickey MJ Johnston B McCafferty DM Raharjo E Huang PL. Neuronal nitric oxide synthase (NOS) regulates leukocyte-endothelial cell interactions in endothelial NOS deficient mice. Br J Pharmacol (2001) 134:305–12. doi: 10.1038/sj.bjp.0704234 PMC1572945 11564648

[135] Hickey MJ Granger DN Kubes P. Inducible nitric oxide synthase (iNOS) and regulation of leucocyte/endothelial cell interactions: Studies in iNOS-deficient mice. Acta Physiol Scand (2001) 173:119–26. doi: 10.1046/j.1365-201X.2001.00892.x 11678734

[136] Ahluwalia A Foster P Scotland RS McLean PG Mathur A Perretti M. Antiinflammatory activity of soluble guanylate cyclase: cGMP-dependent down-regulation of p-selectin expression and leukocyte recruitment. Proc Natl Acad Sci USA (2004) 101:1386–91. doi: 10.1073/pnas.0304264101 PMC337062 14742866

[137] Barber GN. STING: infection, inflammation and cancer. Nat Rev Immunol (2015) 15:760–70. doi: 10.1038/nri3921 PMC5004891 26603901

[138] Hou J Karin M Sun B. Targeting cancer-promoting inflammation — have anti-inflammatory therapies come of age? Nat Rev Clin Oncol (2021) 18:261–79. doi: 10.1038/s41571-020-00459-9 PMC8978805 33469195

[139] Xin Yu J Hubbard-Lucey VM Tang J. Immuno-oncology drug development goes global. Nat Rev Drug Discovery (2019) 18:899–900. doi: 10.1038/d41573-019-00167-9 31780841

[140] Li X Song W Shao C Shi Y Han W. Emerging predictors of the response to the blockade of immune checkpoints in cancer therapy. Cell Mol Immunol (2019) 16:28–39. doi: 10.1038/s41423-018-0086-z 30002451 PMC6318304

[141] Haslam A Prasad V. Estimation of the percentage of US patients with cancer who are eligible for and respond to checkpoint inhibitor immunotherapy drugs. JAMA Netw Open (2019) 2:e192535. doi: 10.1001/jamanetworkopen.2019.2535 31050774 PMC6503493

[142] Brooks ED Chang JY. Time to abandon single-site irradiation for inducing abscopal effects. Nat Rev Clin Oncol (2019) 16:123–35. doi: 10.1038/s41571-018-0119-7 30401936

[143] Bernstein MB Krishnan S Hodge JW Chang JY. Immunotherapy and stereotactic ablative radiotherapy (ISABR): A curative approach? Nat Rev Clin Oncol (2016) 13:516–24. doi: 10.1038/nrclinonc.2016.30 PMC6053911 26951040

[144] Fukumura D Kloepper J Amoozgar Z Duda DG Jain RK. Enhancing cancer immunotherapy using antiangiogenics: Opportunities and challenges. Nat Rev Clin Oncol (2018) 15:325–40. doi: 10.1038/nrclinonc.2018.29 PMC5921900 29508855

[145] Jain RK. Antiangiogenesis strategies revisited: From starving tumors to alleviating hypoxia. Cancer Cell (2014) 26:605–22. doi: 10.1016/j.ccell.2014.10.006 PMC4269830 25517747

[146] Stein S Pishvaian MJ Lee MS Lee K-H Hernandez S Kwan A. Safety and clinical activity of 1L atezolizumab + bevacizumab in a phase ib study in hepatocellular carcinoma (HCC). J Clin Oncol (2018) 36. doi: 10.1200/jco.2018.36.15_suppl.4074

[147] Motzer RJ Penkov K Haanen J Rini B Albiges L Campbell MT. Avelumab plus axitinib versus sunitinib for advanced renal-cell carcinoma. New Engl J Med (2019) 280:1103–15. doi: 10.1056/NEJMoa1816047 PMC6716603 30779531

[148] Rini BI Plimack ER Stus V Gafanov R Hawkins R Nosov D. Pembrolizumab plus axitinib versus sunitinib for advanced renal-cell carcinoma. New Engl J Med (2019) 280:1116–27. doi: 10.1056/NEJMoa1816714 30779529

[149] Wallin JJ Bendell JC Funke R Sznol M Korski K Jones S. Atezolizumab in combination with bevacizumab enhances antigen-specific t-cell migration in metastatic renal cell carcinoma. Nat Commun (2016) 7:12624. doi: 10.1038/ncomms12624 27571927 PMC5013615

[150] Wu X Giobbie-Hurder A Liao X Lawrence D McDermott D Zhou J. VEGF neutralization plus CTLA-4 blockade alters soluble and cellular factors associated with enhancing lymphocyte infiltration and humoral recognition in melanoma. Cancer Immunol Res (2016) 4:858–68. doi: 10.1158/2326-6066.CIR-16-0084 PMC5050160 27549123

[151] Wu X Giobbie-Hurder A Connolly EM Li J Liao X Severgnini M. Anti-CTLA-4 based therapy elicits humoral immunity to galectin-3 in patients with metastatic melanoma. Oncoimmunology (2018) 7:e1440930. doi: 10.1080/2162402X.2018.1440930 29900046 PMC5993498

[152] Baffert F Le T Sennino B Thurston G Kuo CJ Hu-Lowe D. Cellular changes in normal blood capillaries undergoing regression after inhibition of VEGF signaling. Am J Physiol Heart Circ Physiol (2006) 290:H547–59. doi: 10.1152/ajpheart.00616.2005 16172161

[153] Hurwitz H Fehrenbacher L Novotny W Cartwright T Hainsworth J Heim W. Bevacizumab plus irinotecan, fluorouracil, and leucovorin for metastatic colorectal cancer. New Engl J Med (2004) 350:2335–42. doi: 10.1056/NEJMoa032691 15175435

[154] Schrijvers BF Flyvbjerg A De Vriese AS. The role of vascular endothelial growth factor (VEGF) in renal pathophysiology. Kidney Int (2004) 65:2003–17. doi: 10.1111/j.1523-1755.2004.00621.x 15149314

[155] Scappaticci FA Fehrenbacher L Cartwright T Hainsworth JD Heim W Berlin J. Surgical wound healing complications in metastatic colorectal cancer patients treated with bevacizumab. J Surg Oncol (2005) 91:173–80. doi: 10.1002/jso.20301 16118771

[156] Subbiah IM Lenihan DJ Tsimberidou AM. Cardiovascular toxicity profiles of vascular-disrupting agents. Oncologist (2011) 16:1120–30. doi: 10.1634/theoncologist.2010-0432 PMC3228163 21742963

[157] Martin JD Seano G Jain RK. Normalizing function of tumor vessels: Progress, opportunities, and challenges. Annu Rev Physiol (2019). doi: 10.1146/annurev-physiol-020518-114700 PMC6571025 30742782

[158] Werner ER Blau N Thöny B. Tetrahydrobiopterin: Biochemistry and pathophysiology. Biochem J (2011) 438:397–414. doi: 10.1042/BJ20110293 21867484

[159] Coffelt SB de Visser KE. Immune-mediated mechanisms influencing the efficacy of anticancer therapies. Trends Immunol (2015) 36:198–216. doi: 10.1016/j.it.2015.02.006 25857662

[160] Kim RK Suh Y Cui YH Hwang E Lim EJ Yoo KC. Fractionated radiation-induced nitric oxide promotes expansion of glioma stem-like cells. Cancer Sci (2013) 104:1172–7. doi: 10.1111/cas.12207 PMC7656549 23714583

[161] Kevin Leach J Black SM Schmidt-Ullrich RK Mikkelsen RB. Activation of constitutive nitric-oxide synthase activity is an early signaling event induced by ionizing radiation. J Biol Chem (2002) 277:15400–6. doi: 10.1074/jbc.M110309200 11856735

[162] Berbee M Fu Q Boerma M Pathak R Zhou D Kumar KS. Reduction of radiation-induced vascular nitrosative stress by the vitamin e analog γ-tocotrienol: Evidence of a role for tetrahydrobiopterin. Int J Radiat Oncol Biol Phys (2011) 79:884–91. doi: 10.1016/j.ijrobp.2010.08.032 PMC3023840 20950957

[163] Pathak R Pawar SA Fu Q Gupta PK Berbée M Garg S. Characterization of transgenic gfrp knock-in mice: Implications for tetrahydrobiopterin in modulation of normal tissue radiation responses. Antioxid Redox Signal (2014) 20:1436–46. doi: 10.1089/ars.2012.5025 PMC3936502 23521531

[164] Cheema AK Pathak R Zandkarimi F Kaur P Alkhalil L Singh R. Liver metabolomics reveals increased oxidative stress and fibrogenic potential in gfrp transgenic mice in response to ionizing radiation. J Proteome Res (2014) 13:3065–74. doi: 10.1021/pr500278t PMC4053308 24824572

[165] Rodríguez-Ruiz ME Vanpouille-Box C Melero I Formenti SC Demaria S. Immunological mechanisms responsible for radiation-induced abscopal effect. Trends Immunol (2018) 39:644–55. doi: 10.1016/j.it.2018.06.001 PMC6326574 30001871

[166] Alper T. Adding two components of radiosensitization by oxygen. Int J Radiat Biol (1984) 46:569–85. doi: 10.1080/09553008414551771 6335139

[167] Watts ME Hodgkiss RJ Jones NR Fowler JF. Radiosensitization of chinese hamster cells by oxygen and misonidazole at low x-ray doses. Int J Radiat Biol (1986) 50:1009–21. doi: 10.1080/09553008614551421 3539843

[168] Stevens G Joiner M Joiner B Johns H Denekamp J. Radiosensitization of mouse skin by oxygen and depletion of glutathione. Int J Radiat Oncol Biol Phys (1995) 33:399–408. doi: 10.1016/0360-3016(95)00070-F 7673027

[169] Oronsky BT Knox SJ Scicinski JJ. Is nitric oxide (NO) the last word in radiosensitization? a review. Transl Oncol (2012) 5:66–71. doi: 10.1593/tlo.11307 22496921 PMC3323926

[170] Nagane M Yasui H Yamamori T Zhao S Kuge Y Tamaki N. Radiation-induced nitric oxide mitigates tumor hypoxia and radioresistance in a murine SCCVII tumor model. Biochem Biophys Res Commun (2013) 437:420–5. doi: 10.1016/j.bbrc.2013.06.093 23831468

[171] Brüne B. Nitric oxide: NO apoptosis or turning it ON? Cell Death Differ (2003) 10:864–9. doi: 10.1038/sj.cdd.4401261 12867993

[172] Fajardo LF Stewart JR Cohn KE. Morphology of radiation-induced heart disease. Arch Pathol (Chic) (1968) 86:512–9. 5681435

[173] Kong P Christia P Frangogiannis NG. The pathogenesis of cardiac fibrosis. Cell Mol Life Sci (2014) 71:549–74. doi: 10.1007/s00018-013-1349-6 PMC3769482 23649149

[174] Mezzaroma E Di X Graves P Toldo S Van Tassell BW Kannan H. A mouse model of radiation-induced cardiomyopathy. Int J Cardiol (2012) 156:231–3. doi: 10.1016/j.ijcard.2012.01.038 PMC4899611 22340985

[175] Chello M Mastroroberto P Romano R Zofrea S Bevacqua I Marchese AR. Changes in the proportion of types i and III collagen in the left ventricular wall of patients with post-irradiative pericarditis. Vascular (1996) 4. doi: 10.1177/096721099600400221 8861442

[176] Bentzen SM. Preventing or reducing late side effects of radiation therapy: Radiobiology meets molecular pathology. Nat Rev Cancer (2006) 6:702–13. doi: 10.1038/nrc1950 16929324

[177] Seemann I Gabriels K Visser NL Hoving S Te Poele JA Pol JF. Irradiation induced modest changes in murine cardiac function despite progressive structural damage to the myocardium and microvasculature. Radiotherapy Oncol (2012) 103:143–50. doi: 10.1016/j.radonc.2011.10.011 22112779

[178] Schultz-Hector S Trott KR. Radiation-induced cardiovascular diseases: Is the epidemiologic evidence compatible with the radiobiologic data? Int J Radiat Oncol Biol Phys (2007) 67:10–8. doi: 10.1016/j.ijrobp.2006.08.071 17189062

[179] Anscher MS Vujaskovic Z. Mechanisms and potential targets for prevention and treatment of normal tissue injury after radiation therapy. Semin Oncol (2005) 32:S86–91. doi: 10.1053/j.seminoncol.2005.03.015 16015541

[180] Zhao W Robbins M. Inflammation and chronic oxidative stress in radiation-induced late normal tissue injury: Therapeutic implications. Curr Med Chem (2008) 16:130–43. doi: 10.2174/092986709787002790 19149566

[181] Wynn TA. Cellular and molecular mechanisms of fibrosis. J Pathol (2008) 214:199–210. doi: 10.1002/path.2277 18161745 PMC2693329

[182] Berbee M Fu Q Sree Kumar K Hauer-Jensen M. Novel strategies to ameliorate radiation injury: A possible role for tetrahydrobiopterin. Curr Drug Targets (2012) 11:1366–74. doi: 10.2174/1389450111009011366 PMC3311028 20583982

[183] Rabender CS Mezzaroma E Yakovlev VA Mauro AG Bonaventura A Abbate A. Mitigation of radiation-induced lung and heart injuries in mice by oral sepiapterin after irradiation. Radiat Res (2021) 195:463–73. doi: 10.1667/RADE-20-00249.1 PMC8162938 33822229

[184] Herrera FG Bourhis J Coukos G. Radiotherapy combination opportunities leveraging immunity for the next oncology practice. CA Cancer J Clin (2017) 67:65–85. doi: 10.3322/caac.21358 27570942

[185] Twyman-Saint Victor C Rech AJ Maity A Rengan R Pauken KE Stelekati E. Radiation and dual checkpoint blockade activate non-redundant immune mechanisms in cancer. Nature (2015) 520:373–7. doi: 10.1038/nature14292 PMC4401634 25754329

[186] Spiotto M Fu YX Weichselbaum RR. The intersection of radiotherapy and immunotherapy: Mechanisms and clinical implications. Sci Immunol (2016) 1:EAAG1266. doi: 10.1126/sciimmunol.aag1266 28018989 PMC5171206

[187] Garris CS Arlauckas SP Kohler RH Trefny MP Garren S Piot C. Successful anti-PD-1 cancer immunotherapy requires t cell-dendritic cell crosstalk involving the cytokines IFN-γ and IL-12. Immunity (2018) 49:1148–1161.e7. doi: 10.1016/j.immuni.2018.09.024 30552023 PMC6301092

[188] Tian L Goldstein A Wang H Lo HC Kim IS Welte T. Mutual regulation of tumour vessel normalization and immunostimulatory reprogramming. Nature (2017) 544:250–4. doi: 10.1038/nature21724 PMC5788037 28371798

[189] Muntau AC Röschinger W Habich M Demmelmair H Hoffmann B Sommerhoff CP. Tetrahydrobiopterin as an alternative treatment for mild phenylketonukia. New Engl J Med (2002) 347:2122–32. doi: 10.1056/NEJMoa021654 12501224

[190] Ong GH Lian BSX Kawasaki T Kawai T. Exploration of pattern recognition receptor agonists as candidate adjuvants. Front Cell Infect Microbiol (2021) 11:745016. doi: 10.3389/fcimb.2021.745016 34692565 PMC8526852

